# Evolution of Networks for Body Plan Patterning; Interplay of Modularity, Robustness and Evolvability

**DOI:** 10.1371/journal.pcbi.1002208

**Published:** 2011-10-06

**Authors:** Kirsten H. ten Tusscher, Paulien Hogeweg

**Affiliations:** 1 Theoretical Biology and Bioinformatics Group, Department of Biology, Faculty of Science, Utrecht University, Utrecht, The Netherlands; 2 Scientific Computing, Simula Research Laboratory, Oslo, Norway; University of Basel, Switzerland

## Abstract

A major goal of evolutionary developmental biology (evo-devo) is to understand how multicellular body plans of increasing complexity have evolved, and how the corresponding developmental programs are genetically encoded. It has been repeatedly argued that key to the evolution of increased body plan complexity is the modularity of the underlying developmental gene regulatory networks (GRNs). This modularity is considered essential for network robustness and evolvability. In our opinion, these ideas, appealing as they may sound, have not been sufficiently tested. Here we use computer simulations to study the evolution of GRNs' underlying body plan patterning. We select for body plan segmentation and differentiation, as these are considered to be major innovations in metazoan evolution. To allow modular networks to evolve, we independently select for segmentation and differentiation. We study both the occurrence and relation of robustness, evolvability and modularity of evolved networks. Interestingly, we observed two distinct evolutionary strategies to evolve a segmented, differentiated body plan. In the first strategy, first segments and then differentiation domains evolve (SF strategy). In the second scenario segments and domains evolve simultaneously (SS strategy). We demonstrate that under indirect selection for robustness the SF strategy becomes dominant. In addition, as a byproduct of this larger robustness, the SF strategy is also more evolvable. Finally, using a combined functional and architectural approach, we determine network modularity. We find that while SS networks generate segments and domains in an integrated manner, SF networks use largely independent modules to produce segments and domains. Surprisingly, we find that widely used, purely architectural methods for determining network modularity completely fail to establish this higher modularity of SF networks. Finally, we observe that, as a free side effect of evolving segmentation and differentiation in combination, we obtained in-silico developmental mechanisms resembling mechanisms used in vertebrate development.

## Author Summary

An important question in evolutionary developmental biology is how the complex organisms we see around us have evolved, and how this complexity is encoded in their DNA. An often heard statement is that the gene regulatory networks underlying developmental processes are modular; that is, different functions are carried out by largely independent network parts. It is argued that this network modularity allows both for robust functioning and evolutionary tinkering, and that selection thus produces modular networks. Here we use a simulation model for the evolution of animal body plan patterning to investigate these ideas. To allow for the evolution of modular networks we independently select for both body plan segmentation and differentiation. We find two distinct evolutionary trajectories, one in which segments evolve before domains, and one in which segments and domains evolve simultaneously. In addition, the two evolved network types also differ in terms of developmental dynamics. We show that indirect selection for robustness favors the segments first type networks. Furthermore, as a free side effect, these more robust networks are also more evolvable. Finally, we take into account both functional and architectural aspects to determine the modularity of the network types. We show that segments simultaneous networks generate segments and domains in a integrated manner, whereas segments first networks use largely independent modules to generate segments and domains. Finally, although mimicking natural developmental mechanisms was not part of our model design, the segments first developmental mechanisms resembles vertebrate axial patterning mechanisms. This resemblance arises for free, simply from considering segmentation and differentiation in combination.

## Introduction

A major goal of evolutionary developmental biology (evo-devo) is to understand how multicellular body plans of increasing complexity have evolved, and how the underlying developmental programs are encoded in the genome and gene regulatory network (GRN).

Modern evo-devo research shows more and more that a shared developmental toolkit of signaling, adhesion and transcription factor genes are essential for the development of organisms ranging in body plan complexity from cniderians to arthropods and vertebrates [1]–[3]. Therefore the current paradigm is that body plans of increasing complexity are the result of increases in the complexity of regulation of this similar set of genes [1], [2], [4]–[9] combined with increases in the number of variants of certain developmental toolkit genes [10]–[12]. As a consequence, a strong focus in current evo-devo research is on changes in spatio-temporal gene expression patterns and the differences in architecture of the developmental networks generating them.

Network characteristics that are considered key for the evolution of increasingly complex body plans are modularity, robustness and evolvability. It is frequently argued that developmental GRNs are typically modular, i.e. that different functions are performed by largely independent network parts [2], [13]–[16], and that this is the key property responsible for both network robustness and evolvability. The idea is that modularity reduces pleiotropy, allowing for the malfunctioning of or tinkering with network parts involved in one function without producing failure in other functions [2], [13]–[16]. Although this reasoning sounds appealing and intuitively correct, little research has been done to explicitly test the roles and relationships of developmental network modularity, robustness and evolvability in the evolution of complex body plans. Indeed, we argue that it is currently unclear how modular developmental networks are, how such modularity evolves, and how this modularity looks.

Today, only a limited number of developmental GRNs have been studied in considerable detail. These studied networks are mostly involved in the patterning of a single organ or developmental phase, without detailed knowledge on their relationships with the rest of the developmental network [17]–[22]. As a consequence, although these networks have often been claimed to be modular, it is currently hard to fully assess the modularity of developmental networks.

Based on theoretical studies it has been argued that evolution should neither be expected to produce nor to preserve architectural modular networks. This follows from the fact that modular networks form only a small subset of the possible network architectures capable of performing a particular function [23]. Indeed, theoretical studies aimed at investigating the evolution of architecturally modular networks have had to use quite specific fitness targets to obtain modular networks [24]–[27]. On the other hand, it has previously been shown for other genome [28], [29] and network [30] properties that these may arise as a neutral side effect of the mutational dynamics rather than requiring an adaptive explanation. Similar suggestions have been made for network modularity [31], [32].

With regards to the appearance of modularity, note that in its most general sense network modularity is defined fairly functional -different functions are performed by largely independent network parts- but is currently most frequently measured entirely architectural -different modules of genes that are more densely connected with genes within the module than genes in different modules [33]–[35]. However, it is recently being suggested that functional or dynamic rather than architectural network modularity may be most relevant for network functioning and evolution [36]–[40]. Note that architectural and functional modularity do not necessarily overlap. This might mean that different, more functionally oriented methods to measure modularity are needed [36], [37], [40]. Recently, several such methods have been proposed, among which clustering of genes with similar expression in network attractors [36], or with similar knockout effects [40], or with a function in the same specific process [37].

Thus, currently both the extent and shape of developmental network modularity remain unclear. In addition, it is not well known whether evolution of this modularity requires selection for robustness or evolvability or arises neutrally. The goal of the current study is to use computer simulations to investigate what type of network architecture and properties evolve during the evolution of complex body plan patterning. This will allow us to check to what extent evolved developmental networks are modular, whether network modularity is related to increased robustness and evolvability, and what exactly network modularity looks like. In our simulations we select for segmented and differentiated body plans. Segmentation and extensive anterior posterior domain differentiation are considered key innovations of the bilaterian clade, and have been extensively studied both experimentally and theoretically. This will allow us to compare our in-silico evolved developmental networks with actual biological patterning networks and results of previous simulation studies. Furthermore, by independently selecting for segmentation and domain formation we enhance the chances for modular networks to evolve.

We study the different types of evolutionary trajectories that arise, and compare them with respect to network robustness, evolvability and modularity and the type of developmental mechanism they produce. Quite interestingly, we find that there are only two distinct evolutionary strategies to evolve a segmented and differentiated body plan, each resulting in a distinct developmental mechanism. In one strategy, first most segments and only then domains evolve (SF strategy), while in the other segments and domains evolve more or less simultaneously (SS strategy). In addition, we show that in the SF strategy, a complex time transient is responsible for domain differentiation, while a genetic oscillator produces regular body segments. In contrast, in the SS strategy, a complex time transient generates both the body segments and domains. We find that imposed indirect selection for robustness causes the SF strategy to evolve much more frequently than the SS strategy. Furthermore, the SF strategy was also found to be more evolvable.

The different types of expression dynamics involved in segmentation and domain formation, together with the larger robustness and evolvability of SF networks suggests that they may also be more modular. However, frequently used, purely architectural modularity scores suggest that the two network types are equally non-modular. Pruning of non necessary network parts that potentially obscure architectural modularity did not change these results. Furthermore, changing model parameters such that less densely connected networks evolve also did not produce architecturally modular networks. Therefore, we also used a more functionally oriented method. Specifically, we take into account the fact that the networks generate both segments and domains and investigate whether or not there are relatively independent network parts responsible for these two processes. Using this approach we could demonstrate that while SS networks generate segments and domains in an highly integrated manner, SF networks generate segments and domains in a more modular manner.

Our results show that evolved developmental networks are not necessarily highly modular, robust or evolvable. However, upon significant selection for robustness, networks that are more modular, robust and evolvable will dominate. Our results thus confirm the relationship between modularity, robustness and evolvability. Our results also show that the type of modularity that evolved could not be detected by frequently used, automated, purely architectural algorithms, but required a more functionally oriented method. Beslon recently reported similar results [40]. Importantly, these results suggest that for the detection of biologically meaningful modularity purely architectural methods are less suitable and approaches (also) taking into account network dynamics and function should be preferred.

Intriguingly, we find that the patterning mechanism employed by our SF networks shares key characteristics with vertebrate somitogenesis and axial patterning, even though this was not a specific aim of our study or explicit part of our model design.

## Methods

Here we provide a succinct description of the methods used, for a more elaborate description we refer to Text S1.

### General

**Figure 1:**
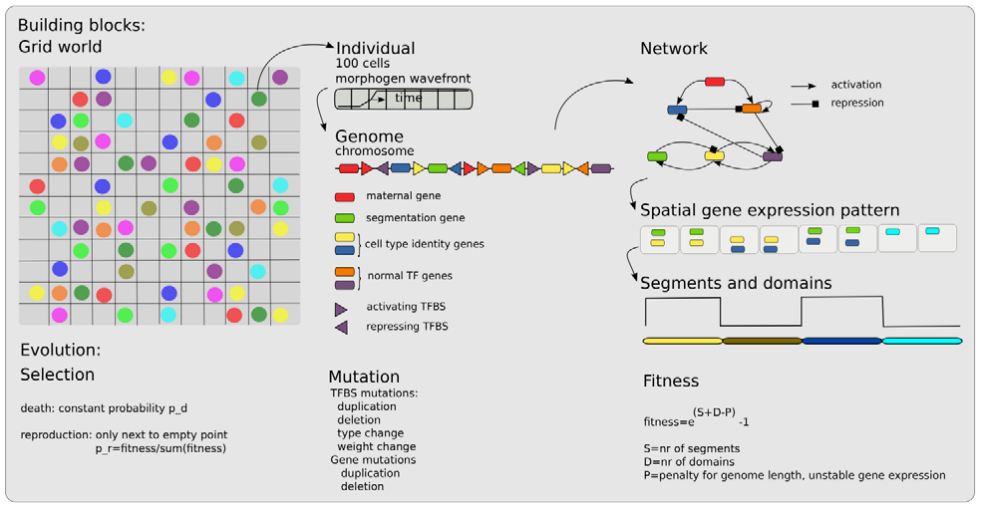
Overview of the model. The in-silico embryos live in a two-dimensional grid world (left). Each individual consists of a one dimensional row of 100 cells over which a maternal morphogen travels to provide some initial spatial information (middle). Each individual has a genome, consisting of genes and upstream transcription factor binding sites (middle) that codes for a gene regulatory network (right). This network dictates the spatiotemporal gene expression dynamics that give rise to the developmental process. The final gene expression pattern is used to determine the number of segments and domains the one dimensional body is divided in by the developmental process (right). An individual's fitness depends on both the number of segments and domains in an independent manner (right). Mutations occur on both genes and transcription factor binding sites (middle). All individuals have the same constant death rate, selection is imposed by making reproduction chances fitness dependent. For more details see text and Text S1.

Briefly, we use an individual based, spatially embedded model of a population of evolving embryo-organisms (Figure 1). The organisms consist of a one dimensional row of 100 cells, similar to the approach followed in [41]–[43]. The organisms have a genome that contains genes coding for transcription factors (TFs) and upstream regulatory regions with transcription factor binding sites (TFBS) [44], [45].

### Segmentation and differentiation genes

Genes have a certain type, indicated with a number ranging from 0 till 15. There can be multiple genes of the same type. The gene types can be subdivided into a few functional categories. Gene type 0 is a maternal gene. Its expression is not controlled by the organism, but instead is imposed to give rise to a morphogen wavefront. This wavefront moves from the anterior to the posterior of the embryo, switching the expression from gene type 0 from a level of 100 to 0. Gene type 5 is a gene that the organisms can use to indicate the boundaries of body segments. Differential expression of gene types 8 till 15 can be used to subdivide the body into functionally different regions (domains). Finally, gene types 1 till 4, 6 and 7 are general transcription factors. By assigning gene type 5 to segmentation and gene types 8 till 15 to differentation the evolving segmentation and differentiation processes are not forced to be coordinated but can in principle use completely disjoint sets of genes.

### Genome, network and development

The genome codes for a gene regulatory network, with genes corresponding to nodes, and TFBS defining the activating and repressing influence of genes on each other. These regulatory links have a non-evolving impact strength of +1 or −1, respectively.

At the beginning of development, gene expression in each cell of an organism is initialized with gene types 1 to 4 having an expression level of 100 and all other genes having an expression level of 0. Subsequent gene expression dynamics and protein levels are governed by the GRN and are modeled with ordinary differential equations using a similar approach as in [41].

### Fitness

The gene expression pattern present at the end of development is used to determine the number of segments and domains the body is patterned in. A segment boundary is defined as a position in space where the expression of the segmentation gene switches from a high to a low level or vice versa. A domain is defined as a region in space where cells express the same combination of differentiation genes at a high level. The minimum length for a segment and domain is 7 cells, allowing for a maximum number of 14 segments and domains. To ensure stable differentiation, we compare gene expression at the end of development with that 20 time steps before. For each cell that has different gene expression levels at these two time points a fitness penalty is applied. In addition, to prevent excessive genome growth small fitness penalties are applied for each gene and TFBS present in the genome (See Table S1 in Text S1).

### Evolution

At the start of evolution the population is initialized with a group of 50 identical organisms in a field of size 30×30. These organisms have a genome containing a single copy of each gene type in a randomized order and with an average of 2 TFBS, randomly drawn from the possible types of TFBS, upstream of each gene. Evolution occurs through mutations on the genome and fitness dependent reproduction. We apply gene duplications and deletions, TFBS duplications and deletions, and changes in the type and weight (activating or repressing) of TFBS. Note that in contrast to some previous studies [41]–[43] we do not evolve gene expression rates, protein decay rates, or TF activation and inactivation threshold levels here. Tournament selection is used to determine which organisms may reproduce. Death occurs with a constant probability of 0.5. After an initial transient population sizes plateau at around 600 individuals.

As explained, we are interested in the robustness, evolvability and modularity of the evolved developmental GRNs. To give evolution the freedom to evolve networks producing segments and differentiation domains either in a modular or integrated manner, we choose our fitness function such the number of segments and domains contribute independently to fitness (i.e. we use 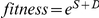 rather than e.g. 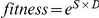). As a side effect of this choice, evolution is also free to evolve only segments or domains, rather than both. For our analysis we select those simulations that were successful in evolving both a significant number (≥7) of segments and domains.

To determine the evolutionary history of a developmental mechanism and its underlying GRN we traced the ancestry of the final fit evolved individuals.

### Simulation experiments and analysis

We performed a total of 50 simulations using the default parameter settings of our model (see Table S1 in Text S1). We analyzed the networks that successfully evolved segmentation and differentiation in terms of evolutionary strategy followed (whether segments and domains evolve sequentially, simultaneously, or something in between), network size and architecture (number of genes and connections, positive feedback loops, attractors) and generated developmental dynamics (type of spatiotemporal gene expression patterns and how this generates segments and domains).

Furthermore we evaluated the robustness, evolvability and modularity of different evolved network types. First, to determine robustness of different evolved network types we performed three additional series of 50 simulations. We increased mutation rate, added gene expression noise, or added variability in morphogen wavefront speed (see Text S1). From the frequency with which the different evolutionary strategies (SS or SF) occur we determine their relative robustness. Second, we performed a total of 140 simulations to find how network types differ with respect to evolvability. Here, we first performed 20 simulations with a fitness target of 6 segments and 6 domains. From these we selected 6 successful networks that differed in type (SS or SF). These were each used as a starting point for 20 independent simulations with a fitness target of 9 segments and 9 domains (see Results). From differences in rates of success of evolving to this second target we determine the relative evolvability of the different network types.

Finally, we determined the modularity of the different network types. Here we used a range of approaches. First, we determined the architectural modularity of the evolved networks using algorithms that try to find the optimal modularity score or Q value for a network. To ensure that our results were not biased by the particular details of the algorithm used, we used two different methods applying different heuristics. The first uses Newman's leading eigenvector method to determine optimal modularity [33], [34], the second method uses a random walk approach to determine Q values [35]. Furthermore, to allow interpretation of the thus found Q values, we determined Q values for not only random and architecturally modular networks, but also for neutrally evolved networks. These neutrally evolved networks serve as a benchmark against which to test whether there is selection for architectural modularity in our simulations.

However, architectural network modularity can easily be obscured by the presence of non-functional or redundant genes and regulatory interactions. Therefore, we pruned the original evolved networks to a minimal essential core network (see Text S1) and also determined Q values for these core networks. Furthermore, architectural modularity may be obscured by the particular model parameter setting used, when these tend to cause the evolution of densely connected networks. To determine whether this was the case, we performed 3 additional series of simulations in which the impact of TFBS deletion rates on modularity was tested. In the first two series, TFBS deletion rates were increased either twofold or fivefold, while all else was kept the same as in the default simulations. In the last series of experiments, a core network with a relatively high Q value was selected from the set of default simulations. This core network was subsequently taken as a starting point for continued evolution simulations with a fivefold higher than normal TFBS deletion rate.

Finally, as an alternative to these automatic, purely architectural methods of determining network modularity, we also assessed modularity in an alternative way. Here we used the core networks as a starting point to determine the minimal networks needed for either segmentation or differentiation alone. To determine how modular a network is we subsequently asses three points. First, we check how well the minimum networks are capable of autonomously reproducing the original segment or domain pattern. Second, we determine how well they can produce one thing (segments) without as a side effect also accidentally producing the other thing (domains). Finally, we assess the amount of overlap between the two minimum networks. Thus, we assess how functionally autonomous and how functionally and architecturally independent these network parts are. The method thus takes into account prior knowledge about network function (they generate both segments and domains) and considers both functional and architectural aspects of modularity. If the minimum segment and domain networks function are good at reproducing either only the original segment or the original domain pattern and contain only a few overlapping genes and connections, we will classify the network as modular. In contrast, Q value based algorithms may fail to detect modularity if modules share not only connections but also a few genes.

## Results

### Two different types of evolutionary trajectories

**Figure 2:**
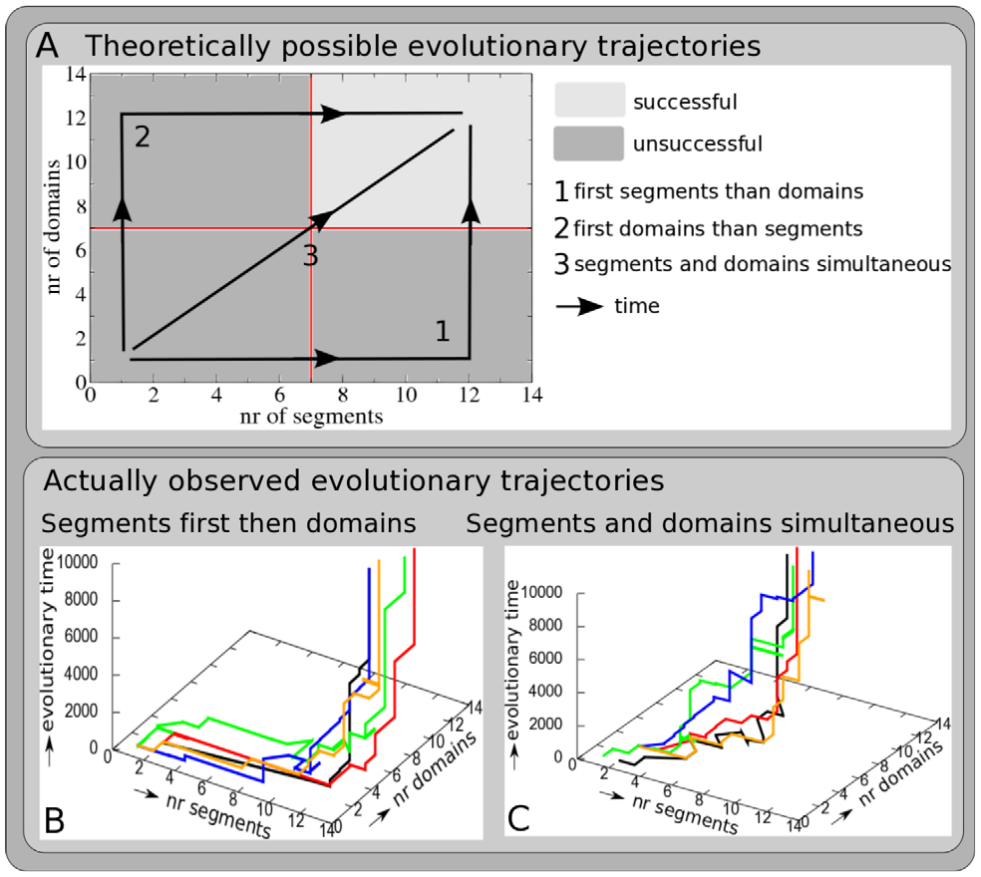
Evolutionary trajectories. **A** The dark and light gray area together form the evolutionary phase plane of possible combinations of segment and domain numbers that can be visited by simulated evolutionary trajectories. If an evolutionary trajectory ends up in the light gray area organisms with 7 or more segments and 7 or more domains have evolved, and the simulation is considered successful. The black lines with arrows indicate the 3 theoretically possible “extreme” evolutionary scenarios: 1) first all segments evolve, then domains evolve; 2) first all domains evolve, then segments evolve; 3) segments and domains evolve simultaneously. In addition, more intermediate evolutionary trajectories may evolve, e.g. sequentially evolving a few segments, a few domains, etc. **B** Example of 5 simulations in which first segments and then domains evolved. **C** Example of 5 simulations in which segments and domains evolved more or less simultaneously.

Figure 2A schematically shows the phase space of possible evolutionary trajectories of evolving both segments and domains. In it we show 3 theoretically possible extreme trajectories: 1) all segments evolve before domains evolve; 2) the opposite, all domains evolve before segments evolve, 3) the intermediate, segments and domains evolve simultaneously.

In our analysis we focus on those 30 simulations (out of the total of 50) in which ≥7 segments and ≥7 domains evolved. We find that in 10 of these simulations (33%) first most segments and then domains evolved. In Figure 2B the evolutionary trajectory of 5 of these simulations is shown. Each trajectory shows the maximum number of segments and domains in the population as a function of evolutionary time. In the 20 other simulations (67%) segments and domain numbers increased more or less simultaneous over evolutionary time. Figure 2C shows the trajectories of 5 of these simulations. None of the simulations first evolved most domains and then segments.

### Network and developmental dynamics of the two evolutionary strategies

A detailed overview of the results of the 10 SF simulations and 20 SS simulations can be found in Tables S4–S9 of Text S1. These results are summarized in Table 1.

**Table 1:** Summary of simulation results.

	SF	SS
**size of evol. network**		
nrgenes original	24.1±3.4	25.5±8.1
nrconn. original	74.7±19.5	96.6±51.7
nrgenes core	19.1±2.7(∼79%)	22.6±8.2(∼89%)
nrconn. core	52.7±14.8(∼71%)	82.9±53.6(∼86%)
**developmental outcome**		
nr of segments	11.7±1.3	8.0±0.8
nr of domains	8.4±1.6	9.3±1.3
**size of min. networks**		
nrgenes minsegm	9.6±1.8	14.5±1.8
nrconn. minsegm	18.9±5.5	35.0±8.7
nrgenes mindom	10.4±3.7	13.4±4.6
nrconn. mindom	16.4±7.6	30.7±16.2
nrgenes sum	15.6±2.4	17.5±2.6
nrconn. sum	30.2±5.3	45.0±12.8
nrgenes overlap	4.4±2.1(∼28%)	10.4±3.6(∼59%)
nrconn. overlap	5.1±3.7(∼16%)	20.7±12.2(∼45%)
**dev. outcome min networks**		
nr of segm minsegm	10.0±1.6(∼85%)	5.9±0.9(∼74%)
nr of dom minsegm	1.4±0.5(∼12%)	4.2±1.8(∼45%)
nr of segm mindom	0	0
nr of dom mindom	3.6±1.4(∼42%; ∼100%)	6.4±2.8(∼68%; ∼98%)
**Q values evol network**		
Qwt original	0.29±0.09	0.30±0.07
Qle original	0.29±0.09	0.27±0.09
Qwt core	0.29±0.08	0.30±0.07
Qle core	0.32±0.07	0.29±0.10
**nr of simulations with osc**	100%	0%

Results shown are for the total of 30 successful simulations in which at least 7 segments and at least 7 domains evolved. Results are subdivided in those of the 10 simulations in which segments evolved first and those of the 20 simulations in which segments and domains evolved simultaneously. Averages and standard deviations are computed. Shown are: 1) the number of genes and regulatory connections in the original evolved networks and their minimum core networks, and how large the core network is relative to the original; 2)the numbers of segments and domains produced by the evolved networks; 3) the number of genes and connections in the minimum segment and domain networks, the sum of unique genes and connections in the two minimum networks together, and the number and percentage of genes and connections overlapping between the two minimum networks; 4) the number of segments and domains generated by the minimum segment network and which percentage this is of the number produced by the original network, the number of segments and domains generated by the minimum domain network, the percentage this is of the number produced by the original network and the percentage this is of the number produced by the core network minus the segmentation gene; 5) Q values found with the walktrap and leading eigenvector methods for both the original and core networks; Finally, the percentage of simulations showing oscillatory dynamics is given.

When comparing network architecture, we find that SF networks are simpler, with similar numbers of genes but significantly lower connectivity. With regards to the network's developmental output, we find that the two alternative strategies attain very similar overall fitness levels. However, SF type networks produce body plans with more segments then domains, whereas the SS type networks do exactly the opposite. In addition, the segments produced by SF networks are much more regularly sized than those produced by SS networks. Indeed, the developmental gene expression dynamics generated by the two network types differ significantly.

**Figure 3:**
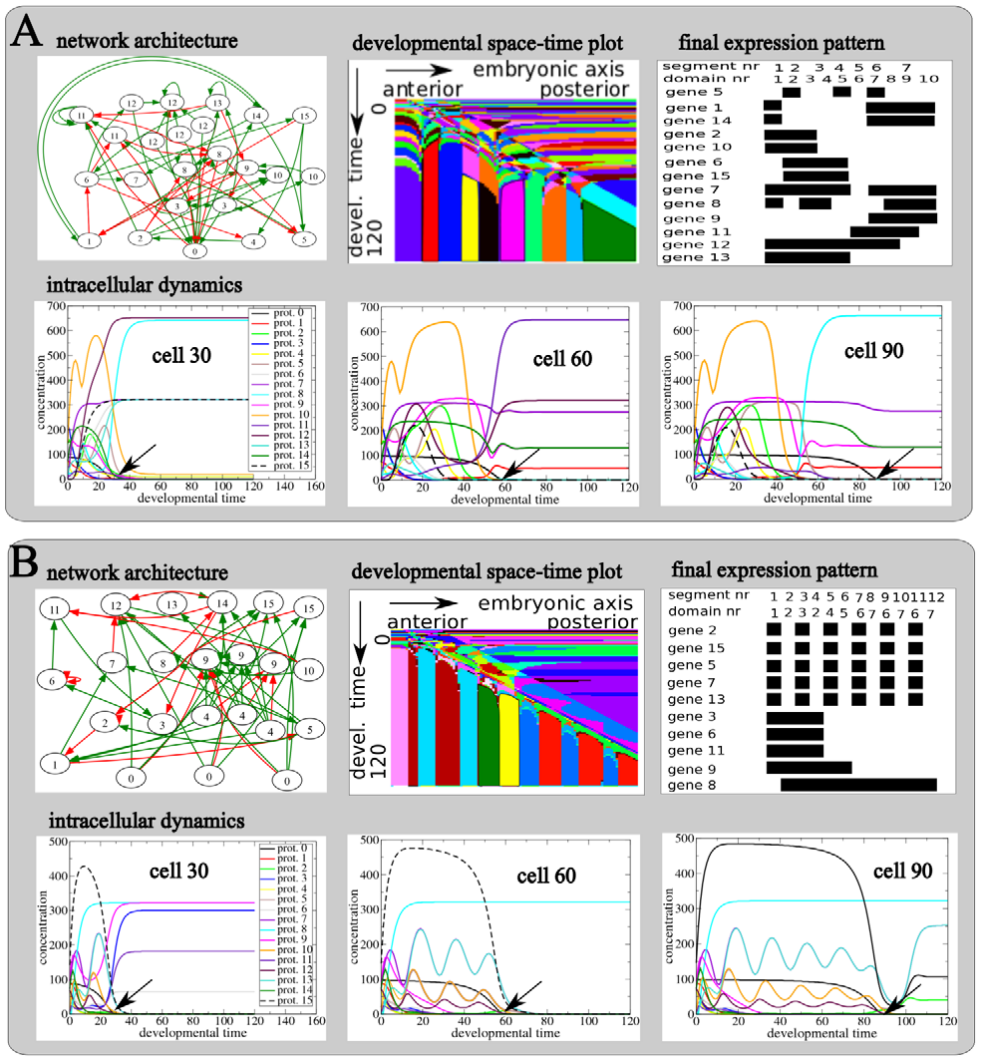
Evolved developmental dynamics. Details of the regulatory network and resulting developmental dynamics for a final fit individual evolved in an example SS (**A**) or a SF (**B**) type evolutionary trajectory. The shown individuals are from the line of ancestry leading up to a fit individual in the final population, and are those individuals in which the final evolutionary innovation occurred. **top row, A and B** Architecture of the evolved gene regulatory network with green activating and red inhibiting interactions; developmental space-timeplot depicting the developmental dynamics produced by the network; and final, end of development gene expression pattern generated by the network. **bottom row, A and B** Detailed temporal protein concentration dynamics produced by the network in cells 30, 60 and 90 along the anterior posterior axis of the embryo. The position of the arrow indicates the time at which the morphogen gradient passes this particular cell.

Figure 3 shows final evolved networks together with the generated intracellular gene expression dynamics, developmental space-time plot, and the final gene expression pattern for both an example SS (Figure 3A) and SF (Figure 3B) network.

We see that the evolved SS GRN is quite complex, containing 24 nodes and 72 connections (Figure 3A, top row). The network produces a complex time transient of gene expression (Figure 3A, bottom row) that upon passage of the maternal morphogen wavefront (gene type 0, arrow) is converted into a stable gene expression pattern. We furthermore observe that the gene types that become stably expressed at a location depend on the time when the wavefront passes. As a consequence, the complex time transient is translated into a temporally stable, but spatially diversified gene expression pattern (Video S1). The space-time plot (Figure 3A, top row) shows another representation of this process. We recognize the anterior to posterior progression of the morphogen wavefront as a distinct diagonal pattern, and see how it transforms the time varying gene expression into a stable spatial pattern (Video S2). If we look at the gene expression pattern at the end of development (Figure 3A, top row) we see that a spatially alternating expression of the segmentation gene (gene type 5) produces 7 body segments of different sizes. The combination of spatially varied expression of the identity genes (gene types 8 till 15) produces a total of 10 domains, also of varying sizes.

The SF network is indeed simpler, containing 23 genes and 57 connections (Figure 3B, top row). The networks produces a complex time transient of gene expression (Figure 3B, bottom row) in which a subset of genes (gene types 2, 5, 7, 10, 12, 13 and 15) display an oscillatory dynamics that we did not observe for the SS network. As for the SS network, the passing by of the morphogen wavefront converts the time-varying gene expression into a stable, spatially varied expression pattern (Video S3, Video S4). However, in this case the oscillatory dynamics of genes 2, 15, 5 and 7 are translated into a regular, alternating expression pattern, allowing gene type 5 (segmentation gene) to produce 12 regularly sized segments (Figure 3B, top row). This mode of producing segments resembles the process of somitogenesis in vertebrates. In addition, the non-oscillatory dynamics of genes 3, 6, 8 9 and 11 are converted to 4 continuous, staggered expression regions (Figure 3B, top row). This expression pattern resembles the typical expression pattern of Hox genes along the anterior posterior axis of bilaterian animals. As genes 8 till 15 all are identity genes, the combination of the alternating expression of gene 13 and 15 and the continuous staggered expression of genes 8, 9 and 11 produce a total of 7 different domains (if multiple regions express the same set of identity genes only the first counts as a domain).

Similar results were found for other SS and SF networks. Thus, while SS networks use a complex time transient to produce both segments and domains, SF networks use a similar complex time transient to produce domains, while using oscillatory dynamics to produce regularly sized segments. In later sections we discuss further details of these developmental dynamics in the context of network modularity.

### Robustness of the two evolutionary strategies

We found that under the default parameter settings (Table S1 in Text S1) the SS strategy evolved more frequently than the SF strategy. Next, we investigated how the propensity of the two evolutionary strategies is affected by adding noise to our simulations. Previous research has shown that robustness evolves as a result of increased mutational or gene expression noise [46]. Here we thus assume that increased noise, independent of the type of noise, produces indirect selection for robustness. By assessing the frequency with which the different strategies evolve under increased noise we investigate which of the two strategies is more robust.

We performed 3 series of 50 simulations. In the first series mutation rate was increased by a factor 10. In the second the propagation speed of the maternal morphogen gradient was varied between individuals within a 30% range. In the third series 5% gene expression noise was incorporated. Table 2 shows the percentage of successful simulations and how often the different evolutionary trajectories were followed. Note that we did not observe any additional types of evolutionary trajectories, i.e. first evolving domains and then segments. We see that for all 3 additional series of simulations a shift occurred from SS as a dominant evolutionary strategy to SF as a dominant evolutionary strategy. Thus indirect selection for robustness favors the SF type networks, suggesting that these are more robust.

**Table 2:** Results of the evolvability test.

simulation series	successful runs	SF	SS
default param. settings	60%	33.33%	66.67%
mutation rate ×10	55%	78%	22%
wavespeed varies 30%	66%	61%	39%
5% expression noise	76%	76%	24%

Shown are the percentage of simulations that are successful (≥7 segments and domains evolved), and the percentage of this subset of successful simulations that evolve using the SF or using the SS strategy. Results are shown for the default parameter settings and for the 3 series of simulations in which indirect selection for robustness was imposed by adding noise. For details on how these 3 additional series of simulations were performed see Text S1.

### Evolvability of the two evolutionary strategies

Next we determined whether the two network types also differed in evolvability. It is frequently thought that a special selection regime is required for the evolution of evolvability [2], [13]–[16]. An often used approach is to impose indirect selection for evolvability by alternating between different selection regimes [44], [47]–[49]. Clearly, such a back and forth alternation between selection criteria is hardly realistic in a developmental context. However, it has been shown that robustness and evolvability of GRNs is strongly correlated [50], [51]. It is thus interesting to investigate whether the differences in robustness we observed between the two evolutionary strategies are correlated with differences in evolvability. Specifically, we tested for differences in the evolutionary potential of the two network types for evolving new segments and domains.

**Figure 4:**
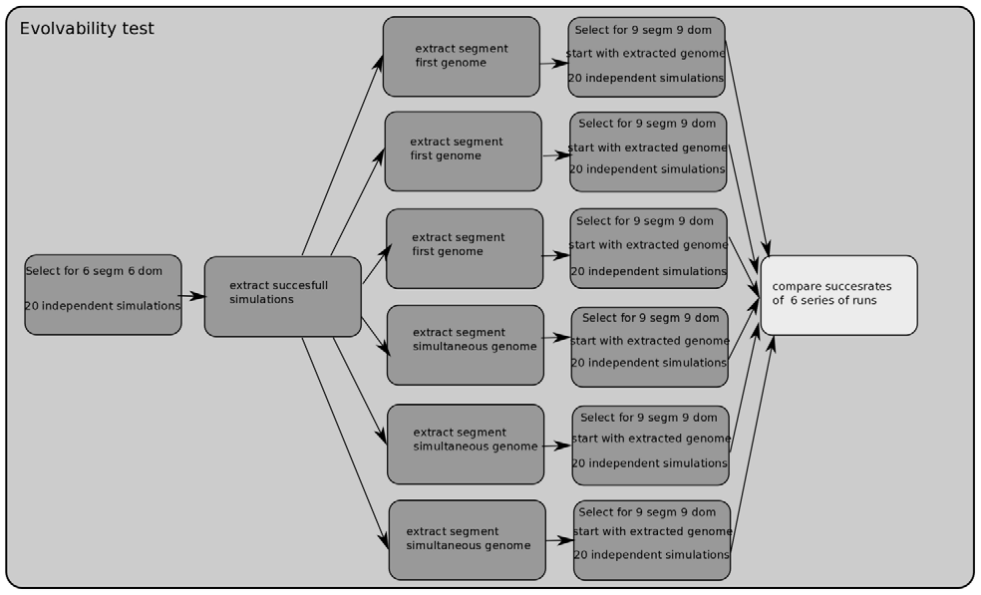
Assessing evolvability potential. Overview of the procedure used to determine differences in evolvability between networks evolved in the different evolutionary trajectories. First, we performed 20 simulations in which we selected for 6 segments and 6 domains. From these 20 simulations we determined the ones that evolved both 6 segments and 6 domains. Next, from these successful simulations, we selected 3 simulations following the segments first and 3 simulations following the segments simultaneous evolutionary strategy. From these 6 simulations we extracted the genome of a finally evolved, fit individual. Each of these 6 genomes were used as input for a series of 20 independent simulations in which now selection for 9 segments and 9 domains was imposed. Finally, we compared the success rates of these 6 series of simulations and whether these differed significantly.

To do this, we first performed 20 simulations in which we selected for 6 segments and 6 domains (Figure 4). From these simulations we selected the successful ones. Next, we selected 3 SF and 3 SS simulations. From these 6 simulations we extracted the genome of a finally evolved, fit individual. Each of these 6 genomes were used as input for a series of 20 independent simulations in which now selection for 9 segments and 9 domains was imposed. Finally, we compared the success rates of these 6 series of simulations (Table 3) and whether these differed significantly (pairwise t-test) (Table 4).

**Table 3:** Results of the evolvability test.

genome	success rate
1, SF	13 (65%)
2, SF	10 (50%)
3, SF	17 (85%)
avg, SF	13.3±3.5 (67%±17.5)
4, SS	5 (25%)
5, SS	2 (10%)
6, SS	2 (10%)
avg, SS	3±1.7 (15%±8.6)

Shown are the number and percentage of simulations that succeed in evolving to the secondary fitness target of 9 segments and 9 domains. Results are split out for the 6 different starting genomes that were derived from simulations successfully evolving to the initial fitness target of 6 segments and 6 domains. For details see Figure 7 and the text.

We see that simulations started with SF type genomes have a considerably higher success rate than simulations started with SS type genomes (Table 3) and that these differences are significant (Table 4). In contrast, simulations started with different genomes but of the same strategy type have much more similar success rates (Table 3), differences being not or hardly significant (Table 4). Differences in success rate are thus not due to random differences between genomes from different simulations, but rather are due to the more fundamental differences between genomes evolved following SF versus SS type evolutionary trajectories. Clearly, genomes evolved in a SF trajectory have a higher evolvability for inventing new segments and domains. These results imply that increased network evolvability can occur as a byproduct of selection for robustness, rather than requiring selection for evolvability itself.

**Table 4:** Significant differences in evolvability.

genomes	1	2	3	4	5	6
1	-	0.350	0.1516	0.0101	<0.0001	<0.0001
2	0.350	-	0.0176	0.1077	0.0049	0.0049
3	0.1516	0.0176	-	<0.0001	<0.0001	<0.0001
4	0.0101	0.1077	<0.0001	-	0.2221	0.2221
5	<0.0001	0.0049	<0.0001	0.2221	-	no diff
6	<0.0001	0.0049	<0.0001	0.2221	no diff	-

P values for pairwise t-test comparison of the success rate for the 6 different genomes are shown. For details see Figure 7, Table 3 and the text.

Note that it remains an interesting question for further research whether other types of evolvability have also increased. Particularly relevant would be whether the ease with which segmentation and differentiation patterns are maintained if embryo size changes, the ease with which celltypes within domains can be changed, or the ease with which segment and domain numbers can decrease are also increased.

### Modularity of the two evolutionary strategies

#### Architectural modularity scores of the evolved networks

**Figure 5:**
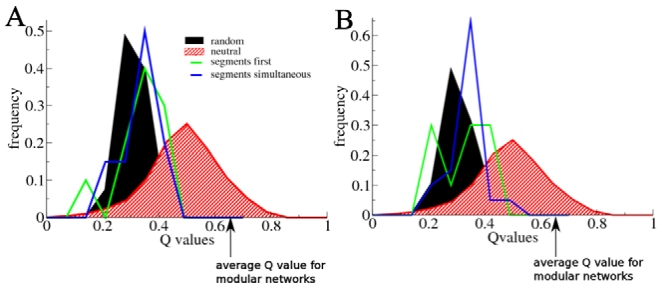
Architectural modularity scores. Q value frequency distributions for random networks, neutrally evolved networks, evolved SF type networks, and evolved SS type networks are shown. In addition, average Q values of manually designed, architecturally modular networks are indicated. Q values shown are those obtained by the walktrap method, for the leading eigenvector method similar values and distributions were obtained (see Tables S4 and S7 in Text S1). For comparison, Q values obtained for modularly designed networks are also indicated. For details on how Q values were obtained see Text S1. **A** Q value distributions for the original, evolved SF and SS networks are shown. For comparison, random networks and manually designed architecturally modular networks of similar size as these original network were taken. **B** Q value distributions for the core networks of the SF and SS networks are shown. For comparison, random networks and manually designed architecturally modular networks of similar size as these core networks were taken.

Next, we determined the modularity scores for both SF and SS networks. Based on the higher robustness and evolvability of SF networks together with the fact that they use distinct expression dynamics to generate segments or domains, one would expect SF networks to be more modular. In contrast, independent of the method used we found for both the SF and SS networks an average modularity score of around 0.29 (see Table 1, Figure 5A and Tables S4 and S7 in Text S1).

#### Setting a baseline for architectural modularity scores

To be able to interpret the meaning of these similar modularity scores, we also determined modularity scores of randomly generated networks, neutrally evolved networks (without a fitness target) and manually designed, architecturally modular networks (see Figure 5 and Text S1). Independent of the modularity algorithm used we found Q values of around 0.29 for random networks (Table S2 in Text S1). For modular networks we found Q scores of around 0.65 (Table S3 in Text S1). Interestingly, for neutrally evolved networks we obtained Q values of around 0.45 (see Text S1). This demonstrates that the mutational dynamics alone causes a significant bias towards architectural modularity, without any present functionality.

If we compare the modularity scores of our evolved networks to these data we see that they are only slightly higher than those of random networks and significantly lower than those of neutrally evolved networks. Thus, selection clearly does not increase the type of architectural modularity measured by the used methods in either the SF or SS networks. This result is further confirmed by the observation that during evolution no significant increases in Q values are observed (see Figure S8 in Text S1).

#### Modularity of core networks and networks evolved with increased TFBS deletion rates

**Figure 6:**
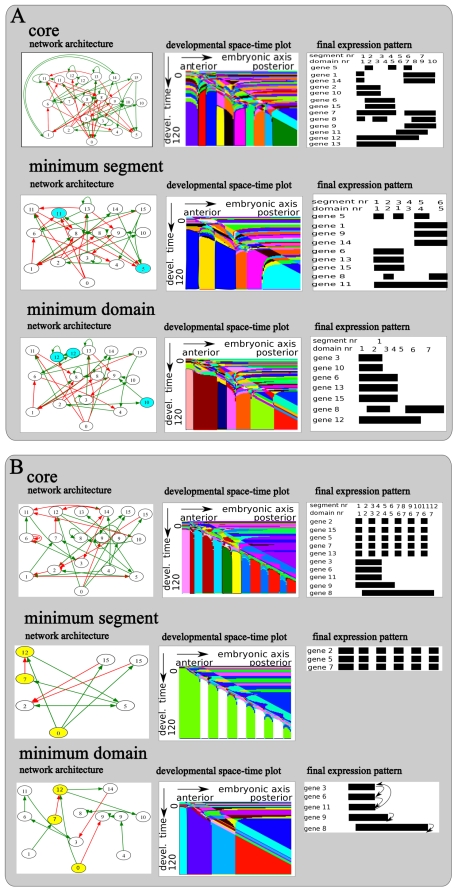
Minimum segment and domain networks. Network architecture, space-time plot of the generated developmental dynamics, and schema of the final produced gene expression pattern for both the core (**top row**), minimum segment (**middle row**) and minimum domain (**bottom row**) networks derived from the example SS (**A**) or SF (**B**) network.

To determine whether non-functional and redundant network parts obscure an underlying architectural modularity we also determined Q values for the core networks derived from the evolved networks. Similar to the original networks, the SF core networks have significantly less connections than the SS core networks (Table 1, also compare Figure 6A and 6B, top rows). However, again Q values of around 0.3 were obtained for both SF and SS type networks (Table 1, Figure 5B, and Tables S4 and S7 of Text S1).

Next, to check whether the parameter setting used causes a bias towards densely connected non modular networks we performed three series of additional simulations in which TFBS were increased. In the first two series we performed simulations that are the same as before, but with two times or five times higher TFBS deletion rates. This did not result in networks with significantly higher architectural modularity scores, independent of whether the original or core networks were evaluated (Table S10 of Text S1). In the final series, we started simulations with a previously evolved core network and continued its evolution under five times higher TFBS deletion rates. As a starting core network we took the core of the SF network shown in Figure 6B, as it has a relatively high Q value compared to average found values. Again, no significant increases in Q values were observed (Table S11 of Text S1). We conclude that frequently used, purely architectural methods to determine network modularity suggest that SS and SF networks are equally non-modular.

#### Alternative evaluation of network modularity

Summarizing, SS networks generate both segments and domains from a complex gene expression time transient, whereas SF networks use a complex time transient to generate domains and oscillating dynamics to generate segments. Furthermore, SF networks are more robust and more evolvable. Still, no differences in network modularity were found using frequently used, purely architectural methods. The question thus is whether SF networks indeed are not more modular than SS networks, or that the methods we used above perhaps fail to uncover certain types of modularity.

Recently, several alternative, more functionally oriented methods to asses network modularity have been suggested. Examples are the clustering of genes with similar expression in network attractors [36], or with similar knockout effects [40], or with a function in the same specific process [37]. Here we also took such a function based approach. We use the fact that networks were evolved to produce both segments and domains, and our observation that SF networks use different dynamics to generate segments or domains. We determine the minimum networks needed for either segmentation or differentiation alone to asses network modularity in an alternative manner (for details see Text S1).

#### SS network

Figure 6A shows the core, minimum segment and minimum domain networks derived from the example evolved SS network, together with the developmental dynamics and final gene expression patterns they generate. The core network has 21 genes and 64 regulatory connections (Figure 6A, top row), and the minimum segment network (Figure 6A, middle) still contains 16 genes and 38 connections. It produces a segmentation gene expression pattern that is shifted relative to the original pattern and capable of producing 6 of the original 7 segments. Furthermore, even though it is only required to produce segments, as a side effect it also produces 5 of the original 10 domains. The minimum domain network (Figure 6A, bottom row) consists of 17 genes and 36 connections. It generates an identity gene expression pattern that is very different from the original, and is capable of producing only 7 of the original 10 domains.

Summarizing, the minimum segment network produces a significant number of domains as a side effect of producing segments, and the minimum domain network performs rather poorly at reproducing the original domain pattern. We conclude that the evolved network is rather non-modular. Instead segments and domains are generated in a highly integrated manner. Indeed, if we compare the two minimum networks, we see that only 2 genes are unique for the minimum segment network and only 3 genes are unique for the minimum domain network (light blue), all other genes are used both for segmentation and domain formation.

Thus, to understand the mechanism behind body plan patterning we should look at the core network, which generates segments and domains in an integrated manner. The observation that a complex gene expression transient is translated into a spatial differentiation pattern suggests two things. First, the core network contains multiple attractors allowing for different stable cell types. Indeed, we see a total of 6 positive feedback loops, essential for attractor formation [52]–[54], in the core network (Figure 6A, top row). Second, the network produces complex and slow expression dynamics, allowing different times of wavefront passage to cause convergence to different attractors. In Text S1 we further explain this developmental mechanism and contrast it with the one described by Francois and Siggia in which a slow timer gene controls a linear sequence of gene activations [43]. Finally, to understand how segments arise as part of this process we study the regulation of the segmentation gene. We see that genes 14 and 15 activate and gene 8 represses gene 5 (Figure 6A, top row). Thus, the spatially alternating expression of gene 5 arises from integrating the inputs of these three genes. Each segment is thus generated by a different combination of regulatory inputs, in a very crude manner resembling Drosophila segmentation.

#### SF network

In Figure 6B we show the core, minimum segment and minimum domain networks derived from the example evolved SF network, combined with the developmental dynamics and final gene expression patterns they generate. We see that, in contrast to the SS network, the core network contains only 18 genes and 36 connections (Figure 6B, top row) and the SF minimum segment network contains only 7 genes and 10 connections (Figure 6B, middle row). The latter produces an oscillatory expression pattern that the passing wavefront transforms into a spatially alternating pattern, producing 11 of the 12 original segments. The segmentation network can be decomposed into a part responsible for generating bistability and a part responsible for producing oscillations, which in combination enable the translation of temporal oscillations into spatial stripes (see Figure S12 in Text S1). Also in contrast to before, the SF minimum segment network does not produce any domains as a side effect.

The minimum domain network (Figure 6B, bottom row) contains 13 genes and 15 regulatory interactions. It produces a complex gene expression transient that generates 4 continuous staggered expression domains, very similar to the Hox-like domains produced by the original network. The SF minimum domain network uses the same developmental mechanism as we discussed before for the core SS network to generate different stable expression domains. In this case, the network contains 3 positive feedback loops: a loop consisting of genes 3, 6 and 11, and positive autoregulation of genes 8 and 9. Further details of this developmental mechanism can be found in Text S1.

However, we also see that the spatially alternating expression of identity genes is not reproduced by the minimum domain network (compare Figure 6B top and bottom row), causing 4 rather than 7 domains to be formed. This shortcoming is due to the standard removal of the segmentation gene from the minimum domain networks (see Methods and Text S1). In the original network the segmentation gene causes genes 13 and 15 to have an alternating expression pattern that contributes to the number of domains. Note however that in contrast to the SS network, the subset of domains that is generated by the minimum domain network corresponds well to those generated by the original network, rather than being shifted in position or expressing different gene combinations.

In contrast to the SS network, the SF minimum networks are thus well capable of generating either segments or domains autonomously and independently. Indeed, the dynamics and expression patterns generated autonomously by the minimum segment and domain networks to a large extent add up to the behavior of the original network. The only clear exception is formed by a subset of identity gene expression domains that are dependent on the segmentation process (see above). However the correspondence is not perfect. For example, the minimum segment number generates a first segment that is too wide and a total of 11 rather than 12 segments (compare Figure 6B top and middle row). In addition, the expression patterns of genes 3, 8, 9, 6 and 11 produced by the minimum domain network are somewhat different than those produced by the original network (compare Figures 6B top and bottom row) (for more details see Text S1). Apparently some of the network parts present in the core network but not in the minimum segment and domain networks are needed both for some segmentation dependent domains and for some additional fine tuning of the segmentation and differentiation processes.

Also in contrast to the SS minimum networks, the two SF minimum networks together contain 17 unique genes, of which only 3 (colored yellow) are shared between the two networks. Together these observations demonstrate that the SF minimum segment and minimum domain networks are modules that are largely independently capable of segmenting and differentiating the body plan. We conclude that the SF network is significantly more modular than the SS network. As discussed above, the SF network is not completely modular: some domains are segmentation gene dependent, some fine tuning between segmentation and differentiation is needed, and a few connections and genes are shared between the minimum networks.

#### Architectural modularity after incorporating prior knowledge

Given the observed modularity of the SF minimum segment and domain networks, we next investigated whether the earlier used purely architectural modularity methods are capable of retrieving this modularity. Put differently, if we sum the minimum segment and domain networks into a single network, do the Q value methods retrieve these modules and assign the summed minimum network a high Q value? Perhaps surprisingly, modularity scores for the summed minimum networks are still lower than those of neutrally evolved networks (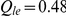 and 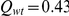, see Text S1). In addition, found architectural modules are inconsistent between the two used methods and unrelated to the above discussed segmentation and differentiation modules (for details see Figure S11 in Text S1).

We suspect that apart from not taking functional aspects into account, an important problem of the architectural modularity algorithms is that even a limited amount of overlap in genes used between functional modules causes them to not be recognized as architectural modules. In contrast, with our alternative method we simply classify a network as being more modular if fewer overlaps between minimum segment and domain networks are found.

Again, similar results were found for other SS and SF simulations (Table 1).

### Sequence of evolutionary innovations in the two evolutionary strategies

As a final part, we investigated whether the differences between the SS and SF evolutionary and developmental strategies are reflected in further differences between their evolutionary dynamics.

#### SS network

**Figure 7:**
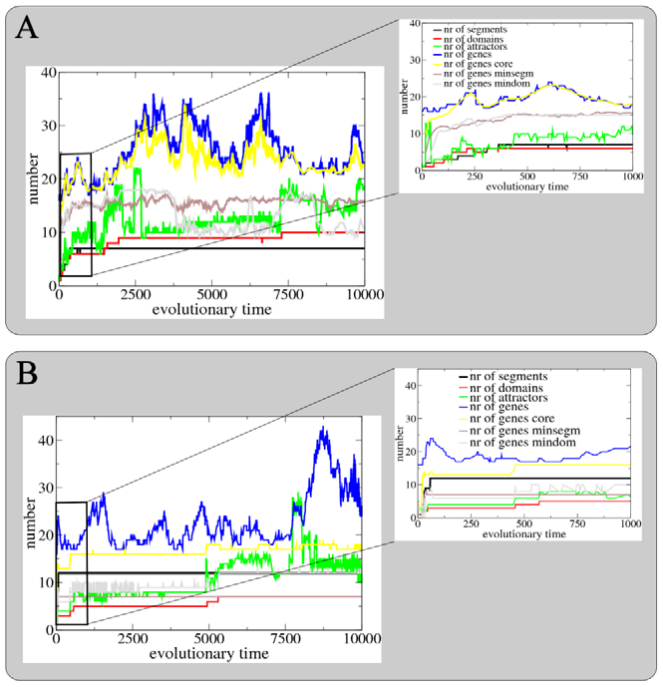
Evolutionary dynamics. Evolutionary dynamics of the number of segments, number of domains, number of network attractors, number of genes in the original genome, number of genes in the core genome, number of genes in the minimum segment genome and number of genes in the minimum domain genome for the example SS (**A**) and SF (**B**) simulations. Numbers are shown for individuals along the line of ancestry. The position of the example SS and SF individuals shown in detail in Figures 3 and 6 is indicated with an arrow. The inset shows in more detail the dynamics up to time 1000.

Figure 7A shows the evolutionary dynamics of segment and domain numbers, attractor numbers and genome size along the line of ancestry leading to fit individuals at the end of the example SS simulation. The arrow indicates the position along this ancestral line of the individual we have analyzed in detail in Figures 3A and 6A. The inset shows the initial phase of evolution. As expected, we see a gradual and simultaneous increase of segment and domain numbers. In addition, the increase in domain numbers appears correlated with an evolutionary increase in GRN attractor numbers. In contrast, we observe no clear correlation between genome size and increases in segment and domain numbers. Instead, genome size shows intermittent periods of expansion and contraction. Interestingly, core genome size is only slightly smaller and has a similar dynamics. This suggests that information is stored in a diffuse, distributed manner, so that when the amount of encoded information (number of segments and domains) increases, the size of the core genome does not change so much.

**Figure 8:**
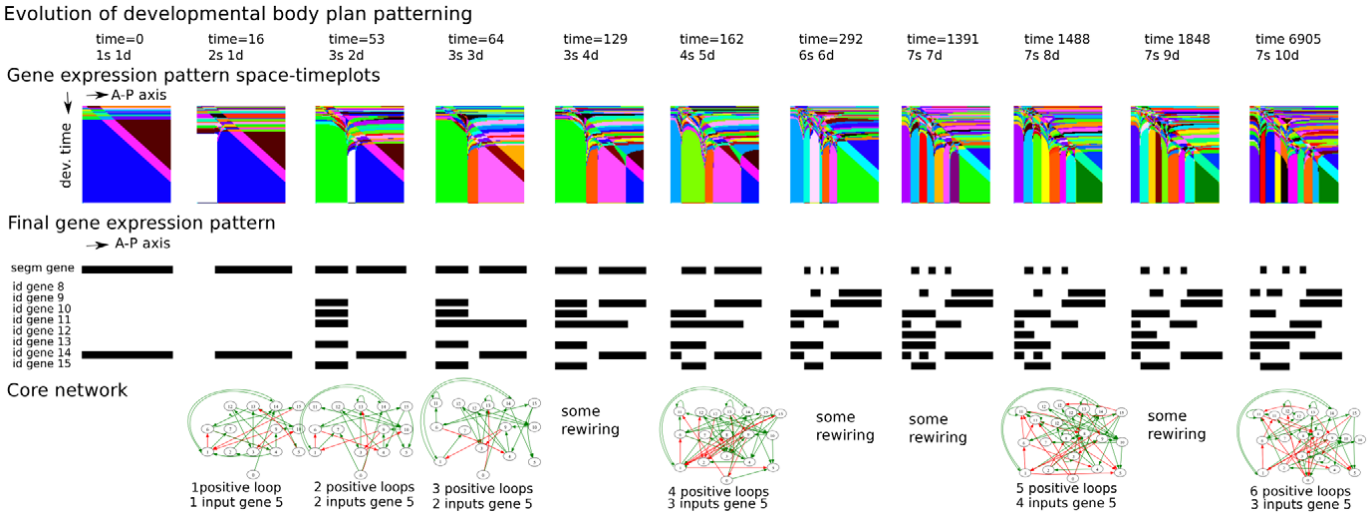
Evolutionary innovations in the SS trajectory. Temporal sequence of the major evolutionary innovations occurring in the example SS simulation (Figures 3, 6 and 7). Shown are the evolutionary time, the number of segments and domains, the developmental space-time plot, the final gene expression pattern, the core gene regulatory network, the number of positive feedback loops and the number of regulatory interactions impinging on the segmentation gene (gene type 5). Only a subset of all evolutionary innovations are shown.

Figure 8 displays another representation of the evolutionary process. Here we depicted those agents along the ancestral lineage in which a major evolutionary innovation arose, i.e. an increase in segment or domain numbers. Note that we only show a subset of selected innovations. As in Figure 7A, we see that segment and domain numbers increase more or less simultaneously and that the number of positive feedback loops present in the core network increases. Furthermore, the number of regulatory inputs to the segmentation gene (gene 5) also increases during evolution. Finally, we see that over evolutionary time there is little conservation of the structure of the core network.

#### SF networks

Figure 7B displays the SF networks evolutionary dynamics. We see the fast initial increase of segment numbers and a subsequent more gradual increase of domain numbers during evolution typical for this type of evolutionary trajectory. As before, the increase in domain numbers is correlated with an increase in attractors. However, we also observe that increases in attractor numbers not always lead to increases in domain numbers (around time 2500). This can be understood from the fact that attractors should be reachable through the developmental process in order to increase domain numbers.

Similar to before, we observe no clear correlation between genome size and increases in segment and domain numbers and instead see intermittent periods of genome expansion and contraction. However, here there is a strong correlation between evolutionary increases in segment and domain numbers and increases in size of the core genome (especially clear in the inset). Similarly, the genome size of the minimum segmentation, respectively minimum domain network are correlated with segment, respectively domain numbers. So, in contrast to what we saw before, here the size of the minimum genome needed to encode the necessary information does increase with segment and domain numbers.

**Figure 9:**
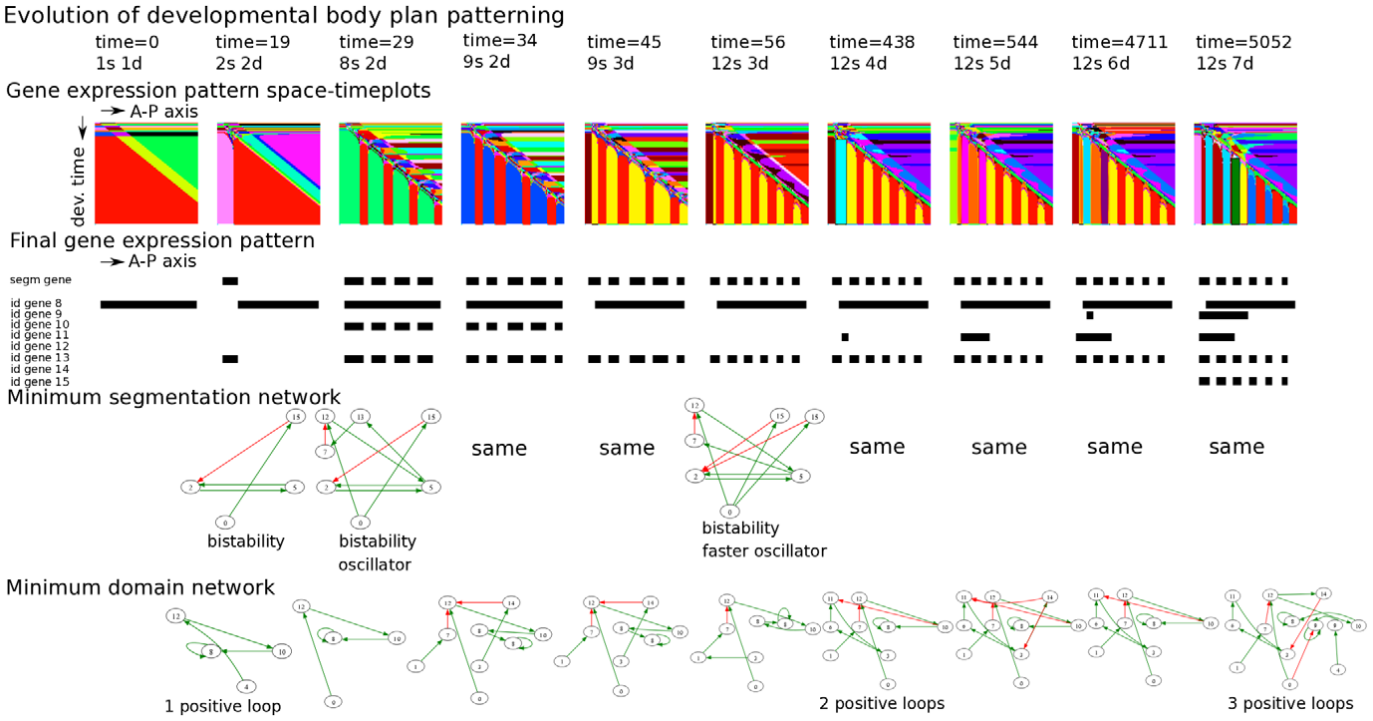
Evolutionary innovations in the SF trajectory. Temporal sequence of all evolutionary innovations occurring in the example SF simulation (Figures 3,6 and 7). Shown are the evolutionary time, the number of segments and domains, the developmental space-time plot, the final gene expression pattern, the minimum segment network and whether it generates bistability or oscillations, the minimum domain network and its number of positive feedback loops.

In Figure 9 we again show those agents along the ancestral lineage in which an innovation arose. We see that first bistability, than oscillations and subsequently faster oscillations are invented (see minimum segment network) generating first 2, then 8/9 and finally 12 segments (see developmental space time-plots). Only later on in evolution the number of domains increases. We can see that part of this increase occurs without the number of positive feedback loops increasing and hence presumably results from network rewiring increasing the independence of already present positive loops (see minimum domain network).

If we compare the minimum segment and domain networks present in the different phases of evolution, we see that previously invented parts are often maintained while new parts are being added. Thus, not only is the final evolved network functionally modular, but these modules are also constructed during evolution in an incremental fashion. This contrasts with the changing nature of the core network we observed for the SS strategy.

## Discussion

In this paper we investigated the in-silico evolution of complex body plans that are both segmented and show anterior-posterior differentiation. An implicit assumption of our study thus is that extensive body plan differentation and segmentation tend to evolutionary co-occur. We base this on the fact that most unsegmented, relatively simple animals such as cniderians possess only a small number of different Hox genes and body domains. In contrast, more complex animals with a larger set of Hox genes and more extensive anterior posterior patterning are either segmented, or show signs of past segmentation [55]–[58]. Note that we made no further assumptions on the order in which segmentation and differentiation evolved, or on whether they evolved once or multiple times [58]–[64].

However, the main aim of the current study was not to settle any of the above issues, but rather to use this setup to study whether or not modular developmental networks evolved. We furthermore investigated how evolution of developmental network modularity depends on indirect selection for robustness. In addition, we studied whether evolved modularity and robustness influence future evolvability. Indeed, we could have used a much more general fitness criterion for body plan patterning, for example maximizing the number of celltypes [65]–[67] or the amount of positional information [68], to study these issues. Instead, we decided to use a more specific fitness criterion that ‘invites’ modularity to evolve, by independently selecting for two functions, segmentation and differentiation. Furthermore, we wished to study segmentation and differentiation as these are considered two major innovations in bilaterian body plan patterning and thus have been extensively studied both experimentally and theoretically.

Evolution was successful in generating body plans that were both significantly segmented and differentiated in 60% of our simulations. This demonstrates two things. First, complex body plan evolution is possible but not trivial. Second, this evolution can be achieved without any coding sequence evolution, by allowing evolution to rewire the regulatory interactions between a simple set of developmental toolkit genes and to duplicate and reuse these genes. Our results thus agree with the argued importance of regulatory evolution [1], [2], [4]–[8] and duplication and divergent usage of existing gene categories [10]–[12] in body plan evolution.

Interestingly, we found that our successful simulations could be divided into only 2 distinct evolutionary scenarios. In 66% of successful simulations segment and domain numbers increased more or less simultaneously during evolution. The evolved developmental networks produced a complex gene expression transient that upon passage of the wavefront was translated into a stable, spatially differentiated expression pattern producing both segments and domains. In the other 33% of successful simulations, first the number of segments increased substantially before the number of domains increased. The evolved SF networks generate gene expression dynamics consisting of a combination of regular oscillations and a complex time transient. The oscillatory dynamics are responsible for producing segments, whereas the complex transient generates domains. Under default parameter settings the segments simultaneous evolutionary strategy is dominant. However, we find that adding noise, thus producing indirect selection for robustness, causes the segments first evolutionary strategy to become the dominant strategy. We furthermore demonstrate that the SF networks also have a higher evolutionary potential for evolving new segments and domains.

Based on the observed differences in expression dynamics, robustness and evolvability we hypothesized that SF networks may also be more modular than SS networks. However, when applying commonly used, purely architectural modularity algorithms similar modularity scores were found for SS and SF networks. Furthermore, these scores were below those of neutrally evolved networks and very close to those of random networks, indicating that no selection for the type of modularity measured by these algorithms occurred.

Only by using our functional knowledge of the networks (they should generate both segments and domains), and taking both functional (different network parts should independently generate either segments or domains) and architectural (these network parts should be largely non-overlapping) aspects of modularity into account could we establish differences in modularity between SS and SF networks. We found that SS networks generated segments and domains in a rather integrated manner, while SF networks operate in a more modular fashion. However, the found modularity was not 100%. Indeed, the SF subnetworks needed to generate either segments or domains share a small subset of their genes and regulatory interactions. Furthermore, a subset of the domains can only be generated in a segment dependent manner. Still, SF networks are considerably more modular than SS networks.

Our results agree with the often heard suggestion that selection for robustness favors modular GRNs and that these modular GRNs tend to be more evolvable [2], [13]–[16]. Furthermore, our findings demonstrate the importance of considering functional aspects of biologically relevant network modularity [36]–[39].

We observed two additional interesting differences between the SS and SF evolutionary strategies. First, while genome size is uncorrelated with body plan complexity for the SS networks, for SF networks not total but core genome size is correlated with organismal complexity. Second, we observed that the complexity and functionality of SF networks changed during evolution in a much more incremental fashion than did the SS networks. Both these differences are likely to contribute to the larger robustness and evolvability of SF networks.

We never observed a domains first segments later evolutionary strategy. In hindsight this is easy to understand. Segments can be generated through two alternative mechanisms. The first, applied in SF networks, uses a segmentation gene oscillator to produce regular segments independent of any domains. The second, used in the SS networks, creates segments by linking segmentation gene expression to the expression of domain forming genes. In this latter case, once a differentiation gene has a spatially varied expression pattern, evolution of a single regulatory link to the segmentation gene suffices to produce segments. Because of this easiness of using domains to make segments, we never observe early evolution of domains with a later evolution of segments.

Previous simulation studies on the evolution of body plan patterning have modeled the evolution of either segmentation [41], [42], [69] or differentiation [43], [65]–[67], [70] alone. The major aim of these studies was to gain an understanding of how natural developmental mechanisms might have evolved. As a consequence these studies focused on the resemblance between in-silico evolved network architectures and those found in nature [41]–[43]. Below we compare our results both to the findings of these earlier studies and to developmental networks found in nature. It should however be kept in mind that in our study this resemblance was neither an explicit aim nor part of our model design.

As discussed above, SS networks generate a single complex gene expression transient that produces both segments and domains. In contrast, SF networks generate both oscillatory dynamics and a complex time transient, the first responsible for producing segments and the second responsible for generating domains. The translation of oscillatory dynamics by a wavefront into a regular segmentation pattern is called the clock-and-wavefront mechanism for segmentation. It was first suggested by Cooke and Zeeman [71] and has been extensively modeled [72]–[76]. This mechanism is responsible for vertebrate somitogenesis [77]–[81], arthropod short germband segmentation and annelid segmentation [64], [82]–[84]. It is suggested to be the ancestral mode of segment formation [60], [62], [85].

Recently, Francois and co-workers [41] found that selection for body plan segmentation in the presence of a propagating morphogen wavefront always leads to the evolution of a clock-and-wavefront type mechanism. In contrast, we find that under selection for both segmentation and differentiation either a clock-and-wavefront type segmentation mechanism or a mechanism in which segmentation depends on the expression of domain forming genes may evolve. In the latter case, segments arise downstream of the differentiation process, with different segments arising from different combinations of domain forming genes. This mechanism very crudely resembles the long germband, Drosophila type of segmentation [86]–[88]. However, in our model segments are formed sequentially rather than simultaneously. The fact that we do not observe a hierarchy of mutual repressors as has been observed in simulations of long germband type patterning [42], [43] is most likely due to this sequential rather than simultaneous patterning. Our results suggest that key to understanding Drosophila segmentation is not just considering that the process occurs simultaneously rather than sequentially, but to also take into account that the segmentation and differentiation processes are tightly integrated.

We found that both SS and SF networks use a complex gene expression transient to produce different domains, and in case of the SS network also different segments. In addition, we found for the SF network that the domains produced were of a continuous staggered nature, somewhat similar to the Hox gene anterior posterior expression domains. In a previous study, Francois and Siggia [43] explicitly selected for such a Hox like differentiation pattern. They found that in case of a propagating morphogen wavefront, a special timer gene was needed to control the order and location in which genes were switched on. The expression level of this timer gene slowly accumulated in the time preceding the passage of the wavefront, thus allowing a translation of wavefront passage time into timer gene expression level and finally expression of a different set of downstream genes. In contrast, in our study we obtained anterior-posterior differentiation without the need for such a timer gene, by combining the presence of alternative attractors with a long and complex time transient. Together this ensures convergence to different attractors at different times of wavefront passage, thus also producing sequential spatial differentation.

Experimental data suggest that the initial Hox gene activation occurring during the primitive streak phase is temporally colinear and may involve timing mechanisms such as chromosomal looping, ordered opening of chromatin domains and cluster level activator and repressor regions [89]–[94]. In contrast, the Hox gene activity in the presomitic mesoderm and during somite formation appears to be under more individual gene level regulatory control [93], [95], [96] and coordinated with the somitogenesis clock and morphogen wavefront [94], [96]–[103]. Indeed, in our segment first simulations we find that the segmentation and patterning processes both depend on the morphogen wavefront (Figure 6B, middle and bottom row), and that they require some coordination (see Figure S10 in Text S1). This resemblance to vertebrate axial patterning evolved for free, as it was neither part of our fitness criterion nor of the model design and is a side effect of considering the combined evolution of segmentation and differentiation. Furthermore, it demonstrates that the evolution of natural developmental mechanisms such as vertebrate axial patterning is neither a very unlikely event nor a completely random outcome, but a type of solution that can be expected.

## Supporting Information

Text S1 **Extended description of the methods and additional results.**
(PDF)

Video S1 **SS spatiotemporal developmental dynamics.** The movie shows the spatiotemporal dynamics of all 16 gene types during development of the example SS individual described in Figure 3A. Gene expression levels (protein concentrations) are encoded in gray scales, white meaning high, gray intermediate and black zero gene expression. The 16 gene types are ordered in 4 rows of 4 genes, running from left top to right bottom. Per gene, the anterior of the embryo is to the left and the posterior to the right.
(MPG)

Video S2 **SS spatiotemporal developmental dynamics -2.** This movie shows again the spatiotemporal gene expression dynamics during development of the example SS individual shown in Figure 3A. Here, in a single plot the expression levels of all 16 genes are drawn as a function of their position along the anterior posterior axis of the embryo, with expression levels changing over time.
(MPG)

Video S3 **SF spatiotemporal developmental dynamics.** Spatiotemporal gene expression dynamics for the example SF individual shown in Figure 3B using the same movie format as in movie S1.mpg.
(MPG)

Video S4 **SF spatiotemporal developmental dynamics -2.** Spatiotemporal gene expression dynamics for the example SF individual shown in Figure 3B using the same movie format as in movie S2.mpg.
(MPG)
