# Supplementary material for: Evolution of Networks for Body Plan Patterning; Interplay of Modularity, Robustness and Evolvability
Source: PLoS Comput Biol. 2011 Oct 6;7(10):e1002208. doi: 10.1371/journal.pcbi.1002208 (PMC3188509; doi:10.1371/journal.pcbi.1002208)
Supplement: Text S1 — Extended description of the methods and additional results. (PDF) [file pcbi.1002208.s001.pdf]

Supplementary Material for: "Evolution of networks for body plan patterning; Interplay of modularity, robustness and evolvability"

Kirsten H. ten Tusscher[1], Paulien Hogeweg[2]
1 Theoretical Biology and Bioinformatics Group, Department of Biology, Faculty of Science, Utrecht University, Utrecht, The Netherlands and Scientific Computing, Simula Research Laboratory, Oslo, Norway
2 Biology and Bioinformatics Group, Department of Biology, Faculty of Science, Utrecht University, Utrecht, The Netherlands
$\ast$ E-mail: Corresponding K.H.W.J.tenTusscher@uu.nl

Extended Methods

Individual-based spatial population model

We use an individual based model of an evolving population of in-silico embryonic organisms living in a two-dimensional grid world (typically 30×30). Each position in this grid world can be either empty or occupied by a single organism. Individual organisms live, move, reproduce and die in this grid world (Figure 1 (left) in article). Movement of organisms is implemented as a random, diffusion-like process [1] (typically 2 diffusion steps per time step), leading to undirected short range displacement of all organisms each time-step. Reproduction is asexual and fitness-dependent. Fitness is computed as a function of the amount of body segments and differentiated domains an organism obtains through its developmental process. Individuals compete to reproduce in an empty grid point with other individuals near this grid point (typically within a radius of 2 grid points) based on their fitness. Organisms die with a constant probability (typically 0.5). Evolution of the population thus occurs through mutation and fitness dependent reproduction.

We use a spatial model as we believe this more closely reflects the locality and discreteness of populations of interacting and competing organisms. In addition, local interactions are more computationally efficient than all against all interactions. However, our results do not depend critically on this model feature.

Individual organisms

Following a similar approach as [2] and [3] our organisms are embryos consisting of a one dimensional row of 100 cells (Figure 1, middle of article). Organisms have an explicit genome that codes for a gene regulatory network (Figure 1 (left and middle) in article). The organisms go through a developmental process. At the start of development, all organisms are initiated with the same gene expression pattern. This expression pattern is the same for all 100 cells, except for the expression level of a so-called maternal gene that acts as a morphogen to generate spatial information in the embryo (see below). The initial gene expression, the expression of the maternal gene and the architecture of the GRN together determine the subsequent gene expression dynamics in each of the 100 cells for a constant number of update steps (typically 120). The gene expression pattern reached at the end of development will be used to determine the final phenotype and fitness of the individual.

Genome and gene functions

Organisms contain a linear genome that consists of genes and their upstream regulatory regions with transcription factor (TF) binding sites (TFBS) (Figure 1 (middle) in article). There is a total of 16 different gene types, numbered from 0 till 15, each coding for a particular protein. Due to gene duplications

(see below) multiple genes of a certain gene type, coding for the same protein, may be present in the genome. Furthermore, we distinguish a number of different categories among these gene types. Note that all genes can act as transcription factors among both genes of the same and genes of other categories.

First, we have a single maternal gene (gene type 0). This gene is not expressed by the embryo itself, rather the protein corresponding to this gene is maternally deposited in the embryo and acts as a morphogen. We use this maternal protein to form a moving wavefront (see Development section). Next, we have a segmentation gene (gene type 5). This gene can be expressed by the embryo itself and serves to demarcate the boundaries of body segments. The location of segment boundaries are defined as a region in space where the expression of the segmentation gene switches from a high to a low level or vice versa (see Figure 1 (left) in article and discussion below).

In addition, we have a total of 8 identity genes (gene types 8 till 15). These genes can be expressed by the embryo itself and can be used to specify different domains. A region of consecutive cells expressing the same set of identity genes at a high level form a single differentiated domain. Different domains are formed by regions expressing different combinations of identity genes. (see Figure 1 (left) in article and the discussion below). Finally we have a total of 6 general TF genes (the remaining gene type numbers), which can function as TF like the above genes, but which do not function in forming a morphogen wavefront, demarcating segments or specifying domains.

At the start of our evolutionary simulations we begin with a population of 50 identical individuals. We generate a genome with a random order of single copies of each of the 16 gene types, and an average of 2 random TFBS upstream of each gene.

Network architecture

The genome codes for a gene regulatory network (GRN), with the genes corresponding to the nodes or vertices of the network and the TFBS corresponding to activating or repressing edges or connections (Figure (left) in article). These edges are directed from the gene coding for a TF protein of a particular type towards those genes that have the matching TFBS in their upstream regulatory region.

Intracellular Dynamics

Intracellular dynamics are described in terms of continuous valued protein concentrations. Each protein is coded for and expressed by a gene of a certain type. There are thus as many protein concentrations as there are gene types. However, as we allow for gene duplications, multiple genes of the same type may determine a single protein concentration.

We model the intracellular protein concentration dynamics as a set of coupled ordinary differential equations, where the intracellular concentration dynamics of each protein X is governed by the following equation, following the approach described in [2]:

$$\frac{dX}{dt} = \sum_{k=1}^{l}(max_{i=1}^{n}(\frac{A_i^2}{A_i^2 + H^2}) * \prod_{j=1}^{m}(\frac{H^2}{R_j^2 + H^2}) * E_{max}) - D * X \quad (1)$$

The first, long part of this equation represents the complex, non-linear integration of activating and repressing inputs at the upstream regulatory region of a gene to determine the expression level of the gene. The second, short part of the equation describes the decay of proteins.

In the first part of the equation k iterates over the different genes of type X coding for protein X, i iterates over the different TF genes A that bind to activating TFBS upstream of a gene X, and j iterates over the different TF genes R that bind to repressing TFBS upstream of a gene X. Note that A_i and R_j are also protein concentrations. Also note that the regulatory status is determined per individual gene of a certain type and summation occurs over these regulatory statuses. Previous theoretical research

has shown that this type of regulatory architecture, in which genes can receive competing activating and repressing inputs, optimizes the information processing capacities of the network [4].

We assumed a sigmoid saturating effect of the concentration of activating (A_i) or inhibiting (R_j) TF proteins on the expression level of protein X. Furthermore, we assume that activation of gene expression is dominated by the most abundant activating TF (the *max* function), whereas repression is assumed to be cooperative, and is hence determined as the product of the present repressing TF (the $\prod$ function). H is the saturation constant (typically put at a value of 60), E_{max} is the maximum expression rate per gene (typically put at a value of 100), and D is the decay rate per protein molecule (typically a value of 0.3 is used).

Development

At the start of development the concentration levels of protein types 1 to 4 are set to 100, whereas the levels of all other protein types are set to 0. Of course, this initial condition is quite arbitrary and similar results can be obtained for different initial conditions.

In addition, we set up a spatial concentration profile of the maternal protein that functions as a morphogen. We use a wavefront that switches expression of the maternal gene from a high (100) to a low (0) value and moves from the anterior to the posterior end of the embryo during development, similar to the approach followed by [2, 5]:

$$M(t,x) = \begin{cases} 100\frac{(x-st)^2}{(x-st)^2+10^2} & \text{if } x > st \\ 0 & \text{else} \end{cases} \qquad (2)$$

with x position (expressed in cell number along the anterior-posterior axis), t is developmental time (running from 0 till 120) and s is wavefront speed (typically 1 cell per time-step). The rationale behind using a morphogen wavefront rather than gradient is as follows. We assume that axial elongation from the posterior end of the embryo is the ancestral mode of development [6–11]. This terminal addition, together with signaling from the posterior end automatically results in a traveling wavefront type of positional information.

Once the initial concentrations for the maternal and other proteins are set, concentrations are updated according to Eq. 1 for a total of 120 steps. In addition the maternal protein concentration is updated according to Eq. 2.

Mutation

Mutations occur on the level of the genome. We have two categories of mutations: gene mutations and TFBS mutations (Figure 1). Gene mutations can lead to the deletion or duplication of a single gene. Gene deletions can only occur if multiple copies of the same gene type are present in the genome, to prevent that a certain gene type can be completely lost from the genome. Upon gene duplication a new copy of the gene is inserted at a random position in the genome. Note that gene duplications and gene deletions are performed on the gene including its upstream regulatory region of TFBS.

TFBS can also be duplicated and deleted. Duplicated TFBS are inserted in the upstream region of a random gene in the genome. In addition, TFBS mutations can change the type of a TFBS (which TF it binds), the weight of a TFBS (whether it activates or represses gene expression). Finally, TFBS innovations allow the generation of a TFBS of any possible type and its random insertion in the upstream region of a gene, independent of whether that particular type of TFBS is still present in the genome (TFBS duplications can only generate TFBS of types that are already present in the genome).

Note that in contrast to other, related studies [2, 3, 5] we only allow duplication or deletion of genes. We do not allow for mutation of the maximum expression level E of the gene, the threshold for half-maximal activation H of the gene by a TF, and the decay rate D of the protein the gene codes for. We

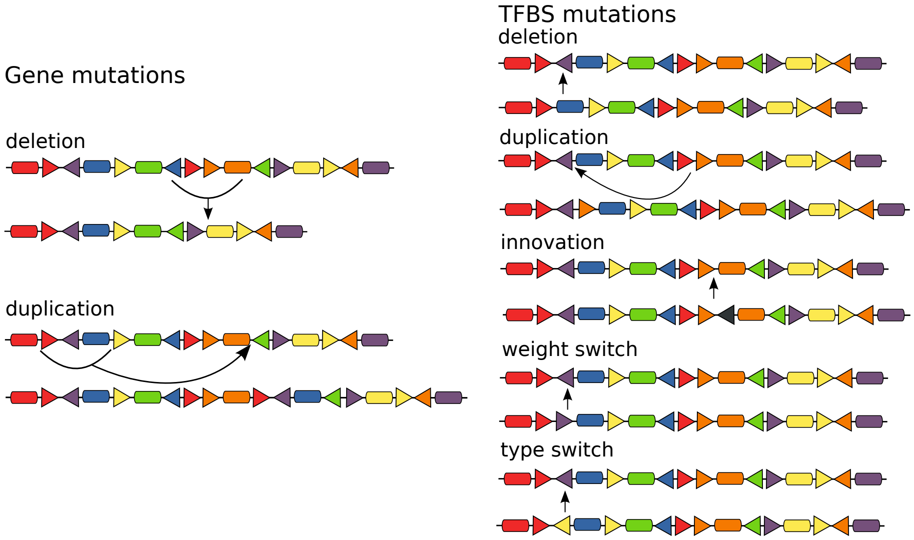

Figure 1. Overview of the different categories of mutations occurring in our model. Mutations on genes lead either to the duplication or deletion of an entire gene including its upstream regulatory region of TFBS. Upon duplication the next copy of the gene is inserted in a randomly drawn position of the genome. On TFBS a larger range of mutations are possible. First, like for genes duplications and deletions of TFBS may occur. Second, TFBS innovations may occur. In this case a new TFBS is created irrespective of whether there is another copy of this type of TFBS present in the genome. Third, the weight (+ or -) of the TFBS, that is is whether it has an activating or repressing effect on the expression of the gene downstream from it, can mutate. Finally, the type of the TFBS, i.e. the type of protein it binds, can change.

choose this approach to reduce the number of parameters that can have different values between different evolved individuals and hence simplify comparisons.

We consider this to be a reasonable simplification for the following reasons. Evolution of different D values allows for proteins with different dynamical time scales, ranging from genes that approach their equilibrium expression level very fast too genes with very slow accumulation dynamics. In a previous study by [5] it was shown that a so-called timer gene that is activated by the morphogen wavefront gene and has a slow timescale of activation allows for the conversion of the moving morphogen wavefront signal into a concentration gradient signal: the level of timer gene activation reached at a particular location is a measure for the time it took for the morphogen to pass that location. The concentration level of the timer gene subsequently leads to spatial differentiation in a manner similar to the spatial differentiation occurring in the presence of a morphogen gradient. Based on these results one could argue that we should allow for the evolution of D values to see whether such a timer gene would arise in our simulations. However, it is important to note that in [5], the authors rationalized how a timer gene could replace a morphogen gradient and then added this timer gene by hand, rather then letting evolution come up with this solution itself. This strongly suggests that it is not quite trivial to evolve this very specific solution. Indeed, if the authors did not put the timer gene in, evolution seemed to come up with different solutions, spreading the timing problem over different genes [5]. It would thus be interesting to see what would happen if the timer gene was put in, but could be lost by evolution, as its full selective advantage is not there straight from the beginning. We therefore consider it highly unlikely for evolution to come up with a timer gene type of solution (if we simply allow D to evolve, rather than putting a timer gene in and preventing its loss) rather than the solutions we did observe in our simulations. Thus, not allowing for evolution of D is not expected to significantly bias our results.

Evolution of different H values for different genes allows the conversion of different input concentration levels into different output expression patterns. Allowing for the evolution of H is clearly essential in the situation where embryos are provided with a spatial morphogen gradient signal, but not in the case where embryos receive a temporal morphogen wavefront signal (assuming that it is not converted into a gradient by using a timer gene, as discussed above). As we were interested in evolution in embryos provided with a morphogen wavefront signal, allowing for evolution of H values was not essential. On the contrary, when playing around with evolving H values, we actually found that this produces artifacts. It turns out that, if H can have any value over a continuous range, even the smallest difference in concentration levels can be converted into a differential expression pattern. However, in real biological systems there are all kinds of noise present. Therefore, for real cells significant differences in expression levels are needed to robustly decide whether or not a gene should be expressed, and the possibility to use infinitesimal differences in concentration to confer positional information in the model is a simple artifact of its determinism and continuity. To prevent these types of artifacts one should allow H values to vary only in discrete steps of a reasonable size and to have a realistic minimum value. Once we did this, only quantitative but no qualitative differences with the results reported here were found.

Selection

Selection occurs on reproduction, whereas death occurs with a constant probability for all individuals. Reproduction of organisms can only occur in empty grid positions. Organisms present within a certain neighborhood of an empty grid point (typically of radius 2) compete for reproduction into this empty grid point. The chance for an organism to produce offspring is:

$$p_{repro}(i) = \frac{f(i)}{\sum_{j \in nb} f(j)} \tag{3}$$

with $f(i)$ the fitness of organism i and $\sum_{j \in nb} f(j)$ the sum of the fitness of all organisms j in the neighborhood of the empty grid point.

The fitness of an organism is defined as follows:

$$\begin{aligned} fitness &= e^f - 1 \\ f &= S + D - P_{inst} - P_{gl} \\ P_{inst} &= \alpha nc_{changed} \\ P_{gl} &= \beta ng + \gamma ntfbs \end{aligned} \quad (4)$$

where S is the number of segments, D the number of domains, P_{inst} a penalty for the number of cells that change their expression pattern during the last 20 steps of development ($nc_{changed}$), and P_{gl} a penalty for genome length (number of genes ng, number of TFBS $ntfbs$). Typically $\alpha = 0.1$, $\beta = 0.00001$ and $\gamma = 0.000001$.

Segments and domains The two most important factors in our fitness criterion are the number of segments and the number of domains an organism divides its 1D body in during development. Segments are repeated, potentially similar structures that allow for a modular buildup of the body plan. Organisms can construct segments by using the differential spatial expression of the segmentation gene to demarcate segment boundaries (see Segmentation section). In contrast, domains provide different regions of the body with a unique identity, allowing for specialization of the different regions. Organisms can construct domains by using the differential spatial expression of identity genes that create regions consisting of different cell types (see Celltypes and Domains section). Segments, cell types and domains are all determined from the gene expression patterns at the end of development.

Additivity Note that in the fitness criterion that we use, the contribution of the number of segments and domains to organismal fitness is summed. An obvious alternative would be to make segments and domains contribute in a multiplicative (i.e $f = e^{S \times D - P_{inst} - P_{gl}} - 1$) rather than additive manner to fitness. In case of the additive fitness function segments and domains can contribute to fitness independently, whereas with a multiplicative fitness function the contribution to fitness of segmentation depends on that of differentiation. The reason for formulating the fitness function in an additive manner is twofold. First, if we use a multiplicative function, we force the evolutionary process to evolve segments and domains simultaneously. In case of the additive criterion the evolutionary process has considerably more freedom and can evolve segments and domains simultaneously, first segments and then domains, first domains and then segments, only segments or only domains. It is a well known fact from other simulated evolution studies that the evolutionary process obtains the best and most interesting results under conditions in which it is the least constrained.

Second, and more specific for the current study, we are here interested in the potential evolution of network modularity, and whether such modularity arises at only the functional or also the architectural network level. The idea behind why network modularity would evolve is that it increases robustness and evolvability by reducing pleiotropic interactions between different network parts and functions. The underlying assumption here is that these different functions contribute at least partly independently to organismal fitness. To understand this consider the following: Assume that a network is functionally modular, but that the fitness contributions of the different functions is not independent, and that a mutation occurs resulting in the failure of one functional module. In this case, the functional modularity will still help in keeping other modules functional (i.e. only S goes down but not D), but this will help significantly less in maintaining a reasonable fitness as it affects the fitness contributions of the still functioning modules. Thus, under the multiplicative criterion a modular network may still maintain somewhat higher fitness values than a non-modular network, but the advantage of modularity is far less than would be the case for an additive fitness criterion. Therefore, to enhance the likelihood of evolving modular networks, we choose to use the additive fitness criterion. It would however remain interesting for a future study to investigate whether modularity could also evolve under a multiplicative or a combined, partly additive, partly multiplicative fitness criterion if significant indirect selection for robustness is applied.

Instability penalty The next most important factor in the fitness criterion is the penalty for unstable cell states. By selecting for segments and domains we select for spatial pattern formation in our organisms. However, different types of pattern forming processes may lead to either transient, cyclic, or stable patterns (see for example [3]). The instability penalty serves to select against transient and cyclic patterns and favors stable patterns. The rationale behind this is that once certain patterns are formed during development they are maintained through subsequent developmental and growth phases and that we should hence select not only for the initiation but also for the maintenance of these patterns.

Genome size penalty The final factor in our fitness criterion is a penalty for genome size which serves to maintain genome size within (computationally) reasonable limits. As has been noted by [12] architectural modularity may require more genes, and hence using genome length penalties may lead to a bias towards non-architecturally modular networks. As we want to investigate whether evolution tends to produce modular networks and whether such modularity is reflected on the architectural level, we surely do not want to include such a bias in our model. Therefore, we used extremely small genome length penalties, 0.00001 per gene and 0.000001 per TFBS, compared to the 1 point fitness gain for each new segment or domain that evolution invents. Put differently, per segment or domain more, an individual could afford 100000 extra genes or even 1000000 extra TFBs without any fitness loss. In other words, these small penalties only serve to prevent excessive genome growth but will by no means prevent normal genome growth.

Finally, the -1 in Eq. 3 serves to obtain zero fitness if the organism has zero segments and domains, and the exponential function serves to obtain "X" times larger fitness values for two times larger numbers of segments and domains (ignoring penalties for simplicity), independent of the absolute numbers of segments and domains.

Domains

In our model the fitness of an embryonic organism depends on the number of different domains it divides its body in during development.

A domain consists of an uninterrupted region of cells expressing the same combination of identity genes at a high level, demarcated by regions of cells expressing a different combination of identity genes. In other words, a domain is formed by a region of cells of a similar type, and different domains consist of different celltypes. This means that we need to determine and distinguish different celltypes.

In boolean type GRNs the state of a cell can be easily typified using a binary code that represents which gene types are switched on (1) or off (0). As a consequence, cells are simply of a different type if they have a different binary code representing their state, and determining the number of celltypes is a trivial task. In continuous state GRNs cell state and cell type are far less trivial concepts due to the continuous range of expression levels. How similar should the gene expression pattern of different cells be to consider them belonging to the same cell type? Is a large differences in the expression of a single gene more differentiating than a collection of smaller differences in the expression of multiple genes? Furthermore, if we choose an arbitrarily sized difference in expression to indicate that cell types are different, across cell gradients in expression levels may arise in which each cell is not different enough from its neighboring cell to be called a different celltype but cells from opposing ends of the gradient are different enough to belong to different celltypes.

Here we tackle this problem using the following approach. First, we defined two threshold protein expression levels, $ThOn$ (typically 80) and $ThOff$ (typically 20). If a protein concentration is above $ThOn$, the corresponding gene is considered (well) expressed. If a protein concentration is below $ThOff$, the gene is considered hardly or not expressed. We then define two cells to be of a different type if there is at least one gene that is well expressed in one of the cells and not or hardly expressed in the other cell.

Another problem we need to consider when determining whether cells are of a different type or not is that a cell type can be characterized by a cyclically varying rather than a constant gene expression pattern. In this case cells can appear different if they have the same gene expression cycle but are out of

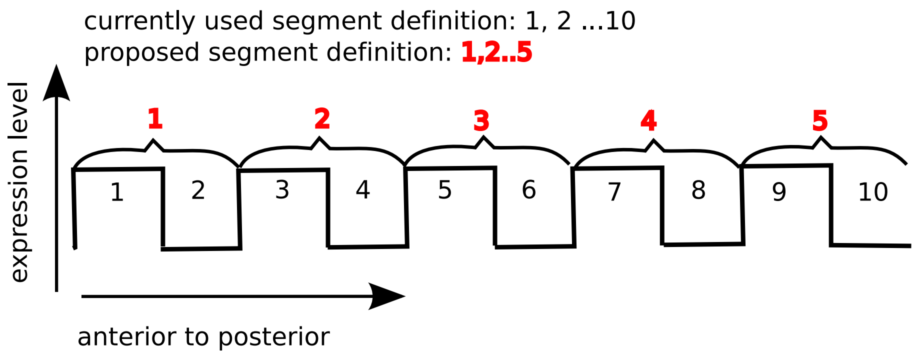

Figure 2. Schematized drawing of how the expression pattern of the segmentation gene is converted into a distribution of segments over the body axis for the current definition and a proposed future definition of segments and segment boundaries

phase. A simple solution to this problem would be to compare time-averaged rather than instantaneous protein concentrations of different cells. Note that here we avoided this problem altogether, at least for the cell types present at the end of development, by explicitly selecting for stable expression patterns (see Fitness section), in order to obtain a stable organismal phenotype at the end of development.

We now define a region as a consecutive series of cells of the same type. Furthermore, we define the number of domains as the number of regions containing different celltypes. Only regions of a minimum length (typically 7), and only regions expressing at least one identity gene are counted as a domain. Regions expressing a different combination of identity genes as the neighboring regions but being of the same celltype as another region in the organism are also not counted as a domain.

Segmentation

Fitness of an organism also depends on the number of segments in which it divides its body during development.

We allow organisms to use a single gene type (gene type 5) to demarcate segment boundaries. We count something as a segment boundary if on one side cells have an expression level of protein $5 > ThOn$ whereas on the other side cells have an expression level of protein $5 < ThOff$. To allow for both steep and smoother segment boundaries the cells with different enough protein 5 expression levels need not be adjacent but can be up to 3 cells apart, with intermediate cells having intermediate expression levels of protein 5.

For a segment to contribute to an organisms fitness we demand that the segment has a minimum length (typically 7 cells), shorter segments are not counted.

Figure 2 shows an example expression pattern for gene 5 and a numbering of the segments it represents. In a late stage of our research we realized that by defining segments in this way (which is the same as the definitions used in [2, 3]), individual segments are not all identical, but rather odd numbered and even numbered segments are identical. Therefore, for future research we consider it to be more reasonable to define segments such that all individual segments are identical, which can be simply operationalized by defining as a segment boundary the location where, measuring in the anterior to posterior direction, the expression level of the segmentation gene changes from $> ThOn$ to $< ThOff$ (or exactly the opposite, that is an arbitrary choice). In Figure 2 we show how this would result in an alternative numbering and total of segments produced by the expression pattern.

(Non-)coordination of segments and domains

From the preceding two sections it follows that domains are determined from the expression pattern of identity gene types 8 till 15, whereas segments are determined from the expression pattern of the segmentation gene, gene type 5. Thus, mechanistically, no coordination between segment formation and domain formation is enforced. Furthermore, from the section on selection it follows that the fitness contribution of segments and domains is additive, and hence independent of one another. Thus, also in terms of imposed selection, there is no enforcing of a coordination between segmentation and differentiation.

Still, in our simulations we very often observe that the spatial boundaries of segments and domains coincide (see for example Figure 3 in the article). This coordination apparently arises for free. In the case of segments and domains simultaneous networks we saw that the segmentation gene is controlled by a combination of differentiation genes, and that hence the segmentation gene is expressed in a subset of the different domains. Segmentation thus depends on differentiation, causing it to be aligned with the differentiation domains. In the segments first networks segmentation and differentiation occur somewhat more independent. Still, often the sequential activation and inactivation of differentiation genes to form domains seems to become partly coordinated with the segmentation clock thus timing domain and segment transitions to co-occur. However, here this coordination is not absolutely necessary, and indeed we see that coordination does not always occur.

If we look for example in Figure 3B, we see the following color pattern being formed in the developmental space-time plot: pink red blue red blue green yellow blue(2) red(2) blue(2) red(2) blue(2) red(2). However, if we look at the final gene expression pattern and the counting of segments and domains, we see that there domains are numbered as: 1 2 3 2 4 5 6 7 6 7 6 7, rather than the 1 2 3 2 3 4 5 6 7 6 7 6 7 which one would naively expect based on the observed color pattern. The reason for this apparent inconsistency is that the first red colored region in the developmental space-time plot has a size of less than 7 cells, which is the minimum size for a segment or domain to be counted. Thus, this short first red region is simply ignored in the final phenotype and fitness determination. The reason behind the formation of this short region is that segmentation and domain formation run here slightly out of phase. More specifically, in the pink area, apart from the segmentation gene 5 and the co-varying genes 2, 7, 13 and 15, genes 3, 6, 9 and 11 are expressed. The pink area transforms into the red region as identity gene 8 also becomes expressed, and a few cells after that the segmentation gene and co-varying genes switch off, giving rise to the blue region. If gene 8 had switched on simultaneously with the segmentation gene and its co-varying genes switching off, the pink region would be followed directly by the blue region and the short red region would not be present.

Coloring of developmental space time plots

In developmental space-time plots each point, corresponding to a particular cell along the anterior posterior axis at a particular point in developmental time, is given a color. The assigned color depends on the genes expressed in that cell at that time. The applied procedure was:

$$
\begin{aligned}
s_i &= \left\{ \begin{array}{ll} 0 & \text{if } X_i < ThOff \\ 1 & \text{if } ThOff < X_i < ThOn \\ 2 & \text{if } X_i > ThOn \end{array} \right\} \\
\textit{celltype} &= \sum_{i=0}^{i=15} (s_i 3^i) \\
\textit{color} &= (\textit{celltype} \times 40)\%251
\end{aligned}
$$

where X_i is the expression level of the genes of type i.
This procedure ensures that the same expression pattern consistently gets assigned the same color, independent of the cell, time or individual in which it occurs. At the same time it ensures that very similar expression patterns are assigned very different colors. Finally it ensures that a finite number of colors

(255) suffices despite a tremendously large number of possible expression patterns. Note that this comes at the cost of the possibility of having different expression patterns getting the same color assigned.

Parameter settings

The default parameter settings are described in Table 1

Adding Noise: Indirect Selection for Robustness

We tested in how far different evolutionary outcomes depend on indirect selection for robustness. We imposed indirect selection for robustness by increasing mutation rates (mutational robustness), by adding gene expression noise to the model (gene expression robustness), or by varying the speed of the maternal morphogen wavefront between individuals (developmental robustness) .

Mutational Robustness

We increased all mutation rates described in Table 1 by a factor of 10.

Gene Expression Robustness

We added a simplified description of gene expression noise to the model. We did this as follows: First we determine per gene X its expression level Exp in the absence of noise using the first part of Eq 1. Then we randomly draw the actual expression level Exp' of gene X from a normal distribution with a mean Exp and a variance of $0.1Exp$. Note that this is applied per *gene* X, not per *protein type* X, for which more than one gene could be coding, each one subject to its own expression noise.

Developmental Robustness

All individuals have a morphogen wavefront moving at a constant speed from the anterior to the posterior of the embryo. However, unlike before, now this wavefront speed varies between individuals. At birth we randomly draw a wavefront speed from the range of 0.85 to 1.15 cells per developmental time-step (before it was 1 cell per developmental time-step) for that particular individual.

Evolvability as a side effect

It has often been assumed that the evolution of evolvability requires a direct or indirect selection pressure for greater evolutionary potential. As a consequence, evolution of evolvability has often been studies using back and forth varying environmental conditions or selection targets [13–16]. This is quite a reasonable approach when considering the evolution of a bacterium in an environment with changing food conditions, but is less reasonable when considering the evolution of the number of segments and domains in the body of a multicellular organism, as one does not expect selection to regularly go back and forth in selecting for different numbers of body parts.

However, although traditionally evolvability and robustness were supposed to inhibit one another studies by Wagner and others have shown that evolvability and robustness may actually be correlated [17, 18]. This may be particularly relevant in the context of network modularity, as modularity has been proposed to contribute both to the current robustness and future evolvability of the networks. So, rather than selecting for evolvability and studying how this influences the evolutionary trajectory followed and final network architecture formed, we investigate whether the different network types that we find, which are found to differ in a correlated manner in functional modularity and robustness, also differ in evolutionary potential. Thus, we ask whether increased evolvability may arise as a side effect of functional modularity and the associated increased robustness.

How exactly this was done was already described in the main article.

parameter	default value
grid size	30 by 30
total evolutionary time	10000
death rate	0.5
reproduction radius	2
α (instability penalty per cell)	0.1
β (gene nr penalty)	0.00001
γ (TFBS nr penalty)	0.000001
initial nr organisms	50
initial nr of genes	16
initial avg. nr of TFBS per gene	3
total nr of gene types	16 (gene types 0-15)
nr of maternal gene types	1 (gene type 0)
nr of segment gene types	1 (gene type 5)
nr of identity gene types	8 (gene types 8-15)
nr of normal gene types	6 (gene types 1-4 and 6-7)
nr of cells in organism	100
nr of developmental steps	120
nr of steps checking stability	20
minimum size of segment	7
speed of maternal wavefront	1
$ThOn$	80
$ThOff$	20
H	60
E	100
D	0.3
tfbs duplication rate	0.0015
tfbs deletion rate	0.0040
tfbs type change rate	0.0010
tfbs weight switch rate	0.0010
tfbs innovation rate	0.0010
gene duplication rate	0.0060
gene deletion rate	0.0090

Table 1. Default parameter settings used for the simulations described in the article. Gene duplication and deletion rates are per gene. TFBS duplications, deletions, type changes and weight changes are per TFBS, TFBS innovation rates are per genome. To prevent either gene evolution or TFBS evolution from dominating the dynamics of our model, we aim for similar overall gene and TFBS mutation rates. Therefore, as a single gene usually has multiple TFBS, and more types of mutations occur on TFBS than on genes, TFBS mutation rates are kept somewhat lower than gene mutation rates. Finally, to prevent excessive genome growth, TFBS and gene deletion rates are chosen to be approximately 1.5 times larger than their duplication rates.

Ancestry Tracing

In the present study we not only investigate the architecture and dynamical properties of the GRNs underlying the final evolved developmental programs, but also those of GRNs arising in preceding evolutionary stages. In order to do so we have to determine the lineage of ancestry from a final evolved fit individual back to an individual in the initial population. We then can study individuals along this ancestral lineage to see when and how particular properties evolved.

Note that in the absence of sexual reproduction, ancestry is indeed along a single lineage rather than a bifurcating network along maternal and paternal lines. In this situation, the simplest way of determining the ancestral lineage leading up to final fit individuals is by attaching to each individual a unique identification number and recording parent-child relationships. The result will be a "perfect fossil record". We do this by storing the identity numbers of the ancestors of each individual in an array. This can simply be done by copying upon birth of a new individual the ancestry array of its parent and then appending the identity number of its parent to this array. At the end of the simulation, this array then contains the full ancestral lineage. Next the simulation can be run again, and now each time an individual is born its identity number is compared against the ancestral array of a final fit individual. This way we can store the genome and other details only of those individuals that arose along this ancestral lineage, saving considerable computer time and disk space.

Modularity

Architectural modularity methods

As a starting point for establishing network modularity, we determined architectural network modularity using the frequently applied idea of finding the subdivision of the network in modules that maximizes the modularity score or Q value. We used two alternative methods that use a fundamentally different heuristic to find optimized network Q values. The reason for taking this approach is that it minimizes the chances of the modularity scores we compute being biased by the particular method that we used. The first method that we used is the leading eigenvector method proposed by Newman [19, 20], which computes the modularity score Q by determining the largest non-negative eigenvector of the network modularity matrix that can be derived from the network adjacency matrix. As this method requires a symmetric network adjacency matrix as input, the original network, with directed, activating and repressing interactions needs to be transformed into an undirected and unweighted graph. The second method that we used, uses random walks on the network to detect densely connected subgraphs and thus determine modularity. The underlying idea is that short random walks will tend to stay within the same module. The method was proposed by Pons and Latapy [21]. This method can be directly applied to directed, weighted graphs. We used the leading.eigenvector and walktrap icommunity functions from the igraph library of R (http://igraph.sourceforge.net/) as implementations of these two methods.

A baseline for architectural modularity scores

To enable interpretation of the modularity scores we obtain for the evolved networks, we also determined modularity scores for random network architectures, neutrally evolved network architectures and modularly designed network architectures. To determine the expected Q value for random network architectures we determined the average Q value of 1000 randomly generated networks containing the same number of genes and connections. Figure 3A shows an example random network with 25 genes, 87 connections and $Q_{el} = 0.236$ and $Q_{wt} = 0.211$. We repeated this procedure for a range of different gene numbers and connection numbers, choosing gene and connection numbers we observed for our evolved networks. These results are summarized in Table 2.

To determine the expected Q value for neutrally evolved networks, we randomly generated 500 genomes and hence networks and let these independently evolve under the same mutational operators and muta-

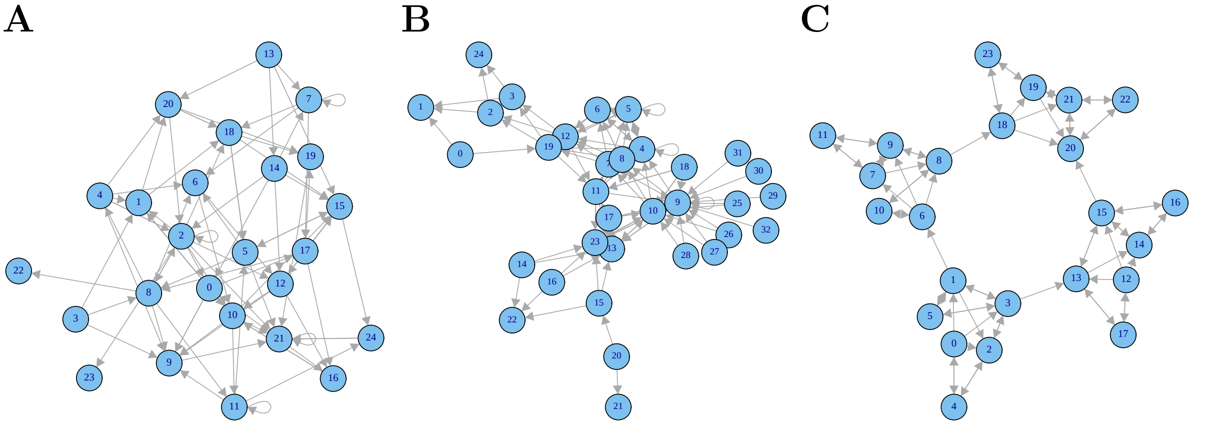

Figure 3. **A** Random network with 25 genes and 87 connections. **B** Neutrally evolved network with 33 genes and 96 connections. **C** Modularly designed network with 24 genes and 84 connections

tional frequencies as normal but without applying any selection pressure.We found an average Q value of around 0.45 for neutrally evolved networks ($Q_{wt} = 0.46 \pm 0.13$, $Q_{el} = 0.44 \pm 0.14$). Figure 3B shows an example neutral network with 33 genes, 96 connections and $Q_{el} = 0.36$ and $Q_{wt} = 0.38$.

Finally, we manually designed a range of modular networks and determined their modularity scores. Figure 3C shows an example modular network with 24 genes, 84 connections $Q_{el} = 0.691$ and $Q_{wt} = 0.691$. We did this for modular networks with a series of different gene and connection numbers, again in the range of gene and connection numbers we observed in our evolved networks. These results are summarized in Table 3.

Avoiding Redundancy: Core Networks

First, we used the architectural modularity algorithms to determine modularity scores of the original, final evolved networks (for results see Result section of this text, Tables 4 and 7). Possibly, there are genes and regulatory interactions in these networks that are non-functional or redundant and only serve to obscure potentially present modularity. Therefore, we also determined the modularity scores of so-called core networks, the minimal networks necessary to produce the original developmental dynamics and final phenotype (for results see Result section of this text, Tables 4 and 7).

Specifically, core networks are defined as the minimum networks still capable of generating the same developmental dynamics, final gene expression pattern and organismal phenotype and fitness as the original evolved networks. Core networks are obtained by pruning the original evolved network. As a pruning criterion we demand that the final gene expression pattern of all gene types should be the same between the original network and the core network. Thus, by definition, all genes and connections in the core network produce a phenotypic effect when manipulated or knocked out.

The pruning is applied randomly, that is we randomly remove a gene (including its upstream TFBS) or a TFBS, decide whether with this new genome and network the organism still functions properly, and repeat this procedure until no more genes or TFBS can be removed or until we performed 200 attempts to remove a gene or TFBS. Note that the thus obtained pruned genomes and networks are not necessarily the smallest possible genomes and networks still functioning properly and may also not be unique. It is in principle possible that if genes and TFBS were removed in a different order, a smaller or slightly different pruned network could be obtained. In practice, for different random seeds and hence different removal orders, we most often find the same or very similar pruned networks. A similar approach was followed in [3, 5]

nr genes	nr conn.	avg Q_{wt}	std Q_{wt}	avg Q_{le}	std Q_{le}
25	87	0.248	0.036	0.216	0.039
21	70	0.243	0.039	0.211	0.043
24	72	0.272	0.037	0.239	0.042
21	64	0.258	0.040	0.228	0.045
23	57	0.315	0.053	0.299	0.057
18	37	0.300	0.042	0.273	0.048

Table 2. Averages and standard deviations of modularity scores obtained for random networks of varying sizes obtained using the walktrap and leading eigenvector methods. Line 1: 25 genes and 87 connections, the average number of genes and connections in the simulated original networks at the end of the evolutionary simulations. Line 2: 21 genes and 70 connections, the average number of genes and connections in the core networks of the networks obtained at the end of the evolutionary simulations. Line 3: 24 genes and 72 connections, the number of genes and connections in the original network shown in Figure 3A for the example segments simultaneous type final evolved network. Line 4: 21 genes and 64 connections, the number of genes and connections in the core network shown in Figure 3B. Line 5: 23 genes and 57 connections, the number of genes and connections in the original network shown in Figure 4A for the example segments first type final evolved network. Line 6: 18 genes and 36 connections, the number of genes and connections in the core network shown in Figure 4B.

nr genes	nr conn.	Q_{wt}	Q_{le}
16	32	0.625	0.625
16	36	0.639	0.639
16	44	0.659	0.659
16	52	0.673	0.673
20	68	0.673	0.673
24	84	0.691	0.691

Table 3. Modularity scores obtained for manually designed, four modules containing networks of varying sizes using the walktrap and leading eigenvector methods. First 4 lines, 16 genes, the number of genes in the networks at the start of the evolutionary simulations, and different amounts of within module connectivity and hence connection numbers. 20 genes and 68 connections, approximately the 21 genes and 70 connections that are the average gene and connection numbers of the cores of the final evolved networks. 24 genes and 84 connections, approximately the 25 genes and 87 connections that are the average gene and connection numbers of the original final evolved networks.

Checking Parameter Dependence: Increased TFBS deletion rates

Another possibility that may obscure the possible evolution of modular networks is that model parameters have been chosen such as to generate very densely connected non-modular networks, whereas under different parameter settings less dense and more modular networks evolve. To determine whether this was possibly the case for the default parameter settings used in our model, we tried out the most straightforward possibility: whether increased TFBS deletion rates and hence lower network connectivity would lead to increased architectural modularity.

In total three additional series of experiments were performed. In the first series TFBS deletion rates were increased twofold, whereas in the second TFBS deletion rates were increased fivefold. Note that for default parameter settings (see Table 1) TFBS deletion rates are already 2.7 times as high as TFBS duplication rates. In the third series of experiments, rather than starting evolution from scratch, we started simulations with the core network shown in Figure 6B of the article, and then applied the 5 times higher TFBS deletion rate (for results see Result section of this text, Tables 10 and 11).

Alternative Modularity Method

Above, we measured network modularity in a purely architectural manner, using automatic algorithms. In addition we will also asses network modularity in an alternative manner that takes into account prior knowledge (i.e. that networks function to generate both segments and domains) and uses both functional (different network parts should be able to function largely autonomously) and architectural (these network parts should hardly overlap) criteria. The principle idea is to determine the minimal networks needed in either segmentation or differentiation alone. We then study how well these networks function autonomously and how much overlap there is between these networks. We consider a minimum subnetwork to be functioning well autonomously if it generates most of the segments or domains generated by the original network (see details below), and does not generate domains as a side effect of producing segments (the vice versa can not occur, see details below). We consider a network modular if the two minimum networks function well autonomously and have little overlap.

Determining minimum segment networks

To avoid any potentially occurring regulatory fine tuning between the segmentation and differentiation processes from obscuring their potential functional independence, we use as a pruning criterion for the minimum segment network a similar number of segments as generated by the original network, rather than demanding the exact same segmentation gene expression pattern. For 4 or less segments the number of segments should be exactly the same (i.e. 4), for 5 segments the numbers of segments should be the same or one less than the original (i.e. 5 or 4), and for 6 or more segments the number of segments should be the same or one or two less than the original (i.e. for 6 segments in original, 6, 5 or 4 in pruned is ok).

In Figure 4 we compare the minimum segment networks obtained when using the above explained criterion versus when demanding that exactly the same number of segments as the original network produces should be generated. We perform this comparison both for the example segments first type network of Figure 3B of the article , as for the example segments simultaneous type network of Figure 3A of the article. In the first case, we see that when demanding that the minimum segment network generates exactly the same number of segments as the original network, we obtain a somewhat larger minimum network. The reason for this is that for the ”oscillator clock” to have exactly the right frequency and to have it immediately from the start of development onwards some extra regulatory links and genes are needed, whereas for the ”ticking of the clock” in approximately the right frequency the basic oscillator and bistability network motifs (shown in Figure 12 of this text) are sufficient. In the second case, for the segments and domains simultaneous network, since the expression of the segmentation gene depends on the identity genes, to get exactly the same number of segments as in the original network we obtain a network very similar to the core network except for the genes and interactions not influencing the segmentation gene's expression.

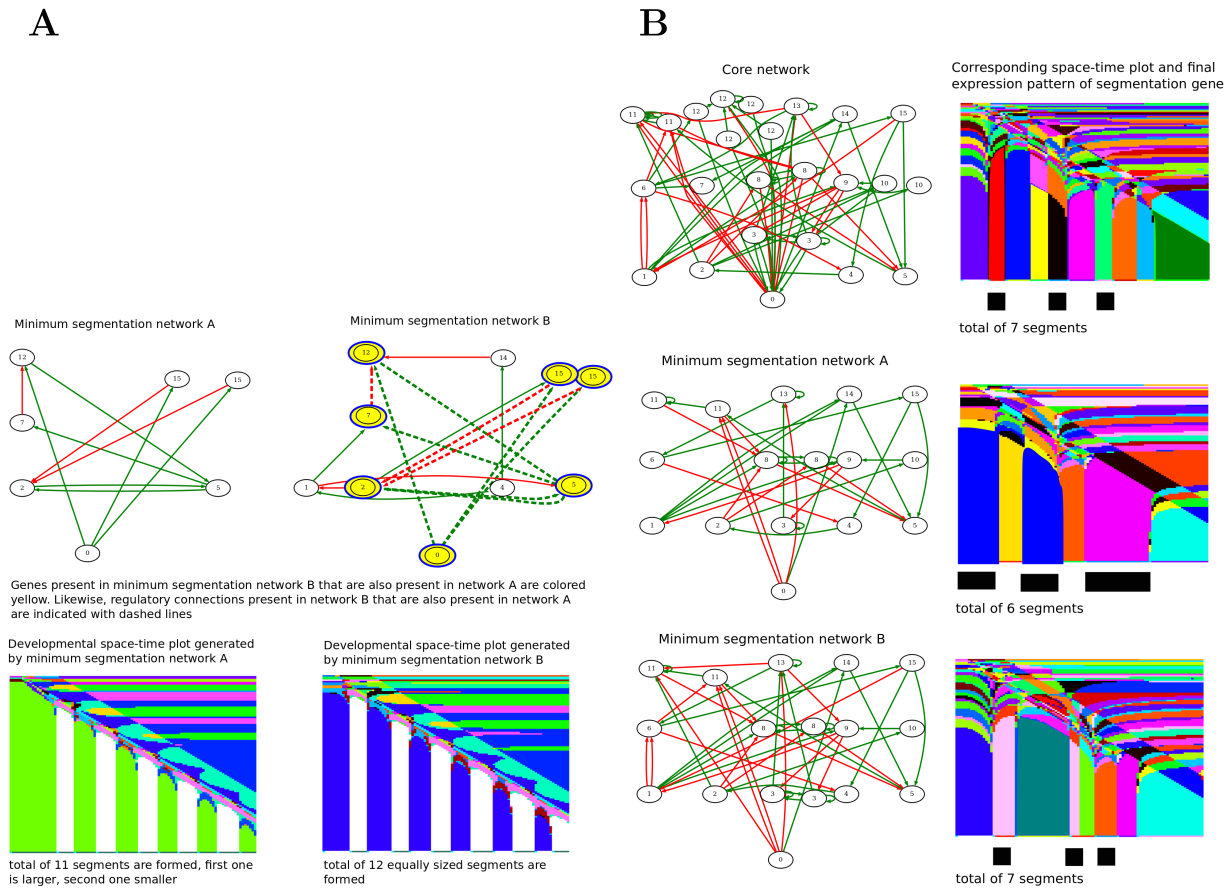

Figure 4. Comparison of two alternative criteria for the minimum segmentation network. Criterion A, the criterion we used throughout our article, demands that the minimum segmentation network should produce the same or a slightly smaller number of segments than the original network. Criterion B, the alternative criterion, demands that the minimum segmentation network should produce exactly the same number of segments as the original network does. We compare both the minimum segment networks that we obtain for the two different criteria, and the developmental dynamics they produce. **A** Minimum segment networks and developmental dynamics for the example segments first type network shown in Figure 3B of the article. **B** Minimum segment networks and developmental dynamics for the example segments simultaneous type network shown in Figure 3A of the article.

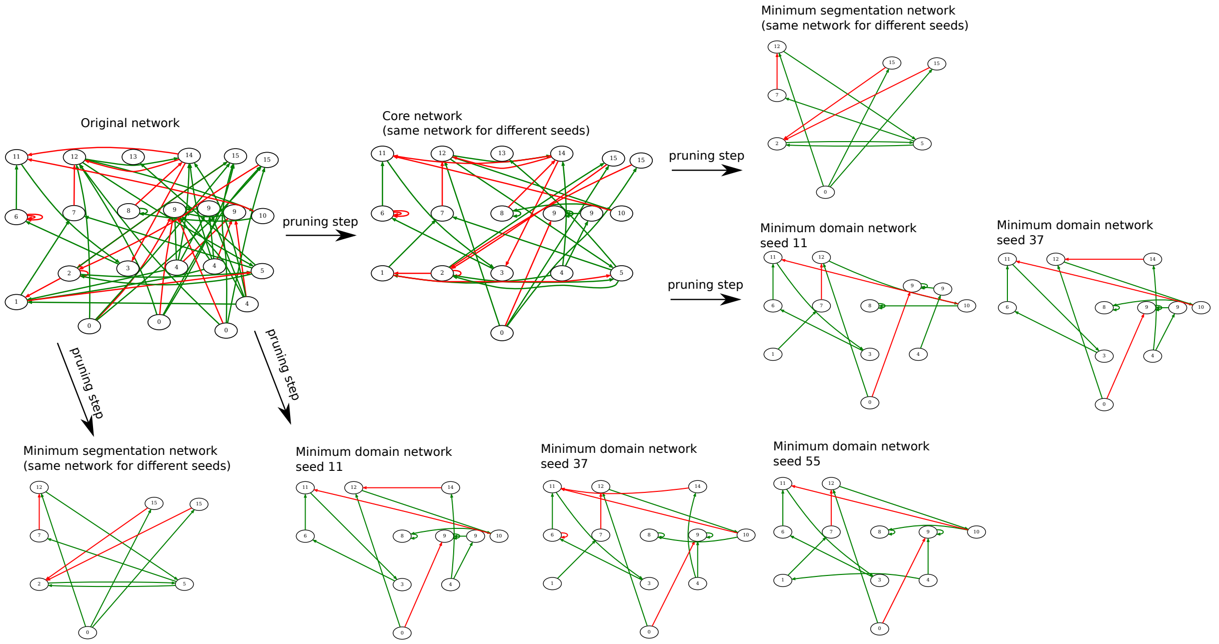

Figure 5. Derivation of minimum segment and minimum domain networks of the example segments first type network of Figure 3B of the article. In the top row, the minimum networks are derived through pruning of the core network, which itself is derived through pruning of the original network. In the bottom row, the minimum networks are derived directly through pruning of the original network. In both cases the pruning is performed multiple times, for different random seeds, possibly resulting in a different order of removal of genes and TFBS and hence final result.

We thus see that a more rigid criterion for the minimum segment networks creates larger networks. Still, for the segments first type networks it still holds that the minimum segment networks are largely independently capable of generating segments. Similarly, for the segment simultaneous networks it still holds that segmentation and differentiation are interlinked (to produce segments you also have to produce domains) and that the minimum networks for segments and domains strongly overlap (now even more so). We choose to use our less rigid criterion as it allows a more clear identification of the bistability and oscillator together producing the basic segmentation dynamics in the segments first strategy.

Determining minimum domain networks Here we again would like to avoid regulatory fine tuning between the segmentation and differentiation processes from obscuring their potential functional modularity. Therefore, to find the minimum domain network we operate as follows. First, we take the core network and remove from it the segmentation gene and its incoming and outgoing regulatory links to remove as much as possible interference from the segmentation process. Next, we determine the new domain pattern formed by this core minus segmentation gene network. We subsequently use this core minus segmentation gene network as a starting point for our pruning procedure to determine the minimum domain network. As a pruning criterion we take that the minimum domain network should generate the same domain pattern as the core minus segmentation gene network.

Starting from the core network

For reasons of computational efficiency we will derive the minimum segment and domain networks by a continued pruning of the core network, rather than starting pruning anew from the original network. In Figure 5 we illustrate the effects of pruning the original versus the core network to obtain minimum segment and minimum domain networks on the example segments first network shown in Figure 3B of

the article. We see that for different random seeds, which possibly generate different random orders in which genes and TFBS are attempted to be removed, always the same minimum segment network is retrieved, independent on whether pruning occurs on the original or the core network. In contrast, slightly different minimum domain networks are retrieved for different random seeds, but the networks found by pruning the original versus the core network are very similar. We find in general that the same or similar minimum segment and minimum domain networks are obtained when pruning is performed directly on the original network as when further pruning is applied on the already pruned core network. We thus conclude that obtaining minimum segment and domain networks from the already obtained core network rather than starting again at the original network does not significantly affect our results.

Architectural Modularity using Prior Knowledge

To see whether architectural modularity methods perform better when taking additional knowledge into account we also assessed the architectural modularity of the summed minimum segment and domain networks.

Positive feedback loop Finding

We wrote a separate routine that finds all positive feedback motifs containing 1, 2 or 3 genes within a network architecture. For reasons of computational efficiency larger positive feedback loops were ignored.

Attractor Determination

We also wrote a separate routine to determine the numbers and types of attractors and the sizes of their basin of attraction. We did this non-analytically, be randomly generating a total of 500000 initial states per network and updating network dynamics until convergence to a attractor occurred. As a consequence, finding all attractors of the network is not guaranteed, but attractors with significant basin sizes are all found with high probability.

Numerical procedures and programming

The simulation code for the model was written in C++. To speed up our simulations the Fast Mersenne Twister algorithm for random number generation (http://www.math.sci.hiroshima-u.ac.jp/ m-mat/MT /SFMT/index.html) was used.

Integration of Eqs 1 and 2 was performed using simple Euler forward integration with a time-step of 1. Admittedly, this time step for Euler integration is on the large side. The reason for using a relatively large time step is to increase computational efficiency, as our simulations are quite computationally demanding (10000 evolutionary time steps, a population of $\sim 600 - 700$ individuals, 100 cells per individual, 16 concentrations per cell, 120 time steps of intracellular dynamics). In Figure 6 we show as an example the gene expression dynamics in cell 15 of the final evolved individual of Figure 5 of the article. We can indeed see that a 10 times smaller time step produces quantitatively slightly different results, but that the qualitative gene expression dynamics and final stable gene differentiation state are unaffected. Note that we choose this relatively simple dynamics as an example on purpose, as for more complex dynamics differences would be even harder to observe.

It should be also noted here that as we are not approximating any real system here but are evolving artificial systems, a very high precision of numerical integration is not particularly relevant as long as all individuals experience the same amount of precision and as long as the system does not come near numerical instabilities producing artifacts (negative concentrations, large and growing oscillations in concentrations, etc). In our simulations, each time protein concentration states are updated we check for

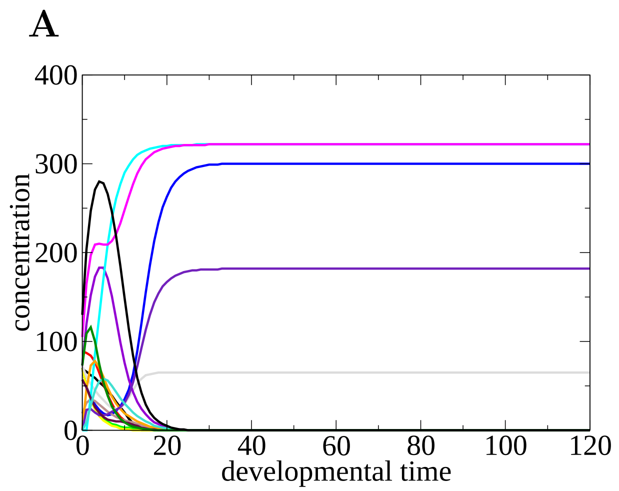

Figure 6. Intracellular dynamics in cell 15 of the individual shown in Figure 4 of the article for different time steps of numerical integration. **A** Intracellular dynamics if a time step of 1 is used. **B** Intracellular dynamics if a time step of 0.1 is used.

these types of artifacts and let the simulation abort if these arise. In all our simulations (a total of well over 400) we never observed any such problems.

Supplementary Results

Overview of Architectural and Alternative Modularity Results

In Tables 4, 5 and 6 we summarize the results of our 20 segments simultaneous type simulations, and in Tables 7, 8 and 9 we summarize the results of our 10 segments first type simulations. Tables 4 and 7 show the number of genes, connections and the walktrap and leading eigenvector modularity scores for the original and core networks of the simulations. Tables 5 and 8 show the number of segments and domains produced by the original (and hence core) networks, the core network minus the segmentation gene and its regulatory effects, the minimum segment network, and the minimum domain network. Also shown is the percentage of segments and domains produced by the minimum segment and domain networks relative to the number of segments and domains produced by the original network. Finally, we show the percentage of domains produced by the minimum domain network relative to the number of domains produced by the core minus segmentation gene network. Tables 6 and 9 show the number of genes and connections present in the minimum segment and minimum domain networks, the number of unique genes and connections present in the two minimum networks together, and the number and percentage of genes and connections overlapping between the two minimum networks.

Modularity of the evolved networks

A baseline for architectural modularity scores

As discussed, we determined modularity scores for random networks, neutrally evolved networks, modularly designed networks and the networks evolved in our simulations. Results of these experiments are described in Tables 2, 3, 4 and 7. In addition, Figure 7 (and Figure 3 in the main article) show the frequency distributions of these modularity scores. We see that on average random networks have a Q

run	orig				core			
	nrg	nrc	Qwt	Qle	nrg	nrc	Qwt	Qle
1	22	72	0.36	0.25	20	64	0.38	0.39
2	37	190	0.32	0.20	37	190	0.32	0.20
3	19	52	0.21	0.25	19	52	0.23	0.27
4	24	48	0.40	0.30	15	34	0.34	0.36
5	30	102	0.29	0.19	23	71	0.29	0.19
6	16	51	0.25	0.23	16	46	0.25	0.23
7	25	11	0.25	0.37	19	59	0.24	0.30
8	19	62	0.17	0.15	19	62	0.17	0.15
9	20	80	0.41	0.42	17	62	0.45	0.45
10	25	152	0.28	0.27	25	152	0.28	0.31
11	22	76	0.32	0.27	22	66	0.32	0.27
12	52	245	0.30	0.28	52	245	0.30	0.28
13	20	48	0.33	0.24	18	44	0.33	0.24
14	21	76	0.15	0.01	20	66	0.15	0.01
15	25	109	0.31	0.32	21	77	0.31	0.32
16	23	73	0.36	0.41	18	47	0.33	0.38
17	29	136	0.29	0.29	21	79	0.33	0.30
18	18	48	0.32	0.29	18	45	0.34	0.35
19	34	118	0.32	0.31	19	47	0.32	0.31
20	26	75	0.34	0.24	20	63	0.32	0.42
avg	25.5±8.1	96.6±51.7	0.30±0.07	0.27±0.09	22.6±8.2	82.9±53.6	0.30±0.07	0.29±0.10

Table 4. Network size and modularity scores for the 20 SS simulations. Per simulation, the number of genes (nrg), number of regulatory connections (nrc) and modularity scores are given for both the original and core networks. Two modularity scores, one obtained with the walktrap (Qwt) and one obtained with the leading eigenvector (Qle) method are shown.

run	original		core-segm		minsegm		mindom	
	nrseg	nrdom	nrseg	nrdom	nrseg	nrdom	nrseg	nrdom
1	7	10	0	7	5 (71%)	5 (50%)	0	7 (70%; 100%)
2	7	11	0	8	4 (57%)	3 (27%)	0	8 (73%; 100%)
3	8	9	0	9	6 (75%)	6 (67%)	0	9 (100%; 100%)
4	9	10	0	10	7 (78%)	3 (33%)	0	10 (100%; 100%)
5	8	11	0	8	6 (75%)	6 (54%)	0	8 (72%; 100%)
6	7	8	0	3	5 (71%)	2 (25%)	0	2 (25%; 67%)
7	9	9	0	9	7 (78%)	6 (67%)	0	9 (100%; 100%)
8	8	11	0	4	5 (63%)	3 (27%)	0	4 (36%; 100%)
9	8	9	0	7	6 (75%)	5 (56%)	0	7 (78%; 100%)
10	8	10	0	9	6 (75%)	3 (30%)	0	9 (90%; 100%)
11	8	7	0	1	6 (75%)	3 (43%)	0	1 (14%; 100%)
12	9	8	0	4	7 (78%)	6 (75%)	0	4 (50%; 100%)
13	9	11	0	11	7 (78%)	6 (54%)	0	11 (100%; 100%)
14	7	9	0	5	5 (71%)	4 (44%)	0	5 (56%; 100%)
15	9	9	0	6	7 (78%)	3 (33%)	0	6 (67%; 100%)
16	8	9	0	8	6 (75%)	7 (78%)	0	8 (89%; 100%)
17	7	9	0	4	5 (71%)	2 (22%)	0	4 (44%; 100%)
18	9	10	0	8	7 (78%)	6 (60%)	0	8 (80%; 100%)
19	7	7	0	5	5 (71%)	3 (43%)	0	5 (71%; 100%)
20	7	8	0	3	6 (85%)	1 (12.5%)	0	3 (38%; 100%)
avg	8.0±0.8	9.3±1.3	0	6.3±2.7	5.9±0.9 (∼74%)	4.2±1.8(∼45%)	0	6.4±2.8(∼68%;∼98%)

Table 5. Number of segments and domains generated by original and minimum networks for the 20 SS simulations. Per simulation, the number of segments and domains produced by the original network, the core network minus the segmentation gene and its incoming and outgoing regulatory links, and the minimum segment and the minimum domain networks are given. For the minimum segment network we also indicate the percentage of segments and domains it produces relative to the original network. For the minimum domain network we indicate the percentage of segments and domains it produces relative to the original network (first number) and relative to the core minus segmentation gene network (second number).

run	segm		dom		sum		overlap	
	nrg	nrc	nrg	nrc	nrg	nrc	nrg	nrc
1	16	39	10	19	10	44	10 (100%)	14 (31.8%)
2	14	27	21	53	23	64	12 (52.2%)	17 (26.6%)
3	15	29	16	40	17	41	14 (82.4%)	28 (68.3%)
4	13	28	12	28	14	32	11 (78.6%)	24 (75.0%)
5	14	33	18	49	20	58	12 (60.0%)	24 (41.4%)
6	13	26	6	9	14	28	5 (35.7%)	7 (25.0%)
7	18	53	18	57	19	59	17 (89.5%)	57 (96.6%)
8	12	29	11	19	16	40	7 (43.8%)	8 (20.0%)
9	14	44	14	47	16	54	12 (75.0%)	37 (68.5%)
10	15	34	18	48	20	56	13 (65.0%)	26 (46.4%)
11	15	29	5	5	15	29	5 (33.3%)	5 (17.2%)
12	18	43	10	13	18	43	10 (55.5%)	13 (30.2%)
13	13	33	16	38	18	44	11 (61.1%)	27 (61.4%)
14	13	34	12	27	16	42	9 (56.3%)	19 (45.2%)
15	14	51	15	37	21	71	7 (33.3%)	17 (23.9%)
16	18	45	15	36	18	47	15 (83.3%)	34 (72.3%)
17	15	37	11	16	16	39	10 (62.5%)	14 (35.9%)
18	12	26	14	30	15	34	11 (73.3%)	22 (64.7%)
19	14	25	14	23	17	36	11 (64.7%)	13 (36.1%)
20	14	35	9	13	16	38	7 (43.8%)	10 (26.3%)
avg	14.5±1.8	35.0±8.7	13.4±4.6	30.7±16.2	17.5±2.6	45.0±12.8	10.4±3.6 (∼59%)	20.7±12.2(∼45%)

Table 6. Size and overlap of minimum networks for the 20 SS simulations. Per simulation, the number of genes and connections in the minimum segment and domain networks, sum of unique genes and connections in the two minimum networks together, and overlap of genes and connections between the two minimum networks are given.

run	orig				core			
	nrg	nrc	Qwt	Qle	nrg	nrc	Qwt	Qle
1	23	57	0.30	0.37	18	36	0.29	0.29
2	28	98	0.34	0.34	21	55	0.37	0.40
3	21	77	0.23	0.34	16	43	0.20	0.29
4	24	61	0.36	0.27	18	45	0.30	0.38
5	31	108	0.25	0.25	18	64	0.19	0.27
6	21	39	0.08	0.07	17	45	0.17	0.18
7	25	95	0.30	0.22	23	72	0.30	0.22
8	25	77	0.31	0.38	22	62	0.26	0.41
9	20	56	0.40	0.39	20	56	0.40	0.36
10	23	56	0.39	0.30	18	41	0.39	0.35
avg	24.1±3.4	74.7±19.5	0.29±0.09	0.29±0.09	19.1±2.7	52.7±14.8	0.29±0.08	0.32±0.07

Table 7. Network size and modularity scores for the 10 SF simulations. Per simulation, the number of genes (nrg), number of regulatory connections (nrc) and modularity scores are given for both the original and core networks. Two modularity scores, one obtained with the walktrap (Qwt) and one obtained with the leading eigenvector (Qle) method are shown.

run	original		core-segm		minsegm		mindom	
	nrseg	nrdom	nrseg	nrdom	nrseg	nrdom	nrseg	nrdom
1	12	6	0	4	11 (92%)	1 (17%)	0	4 (67%; 100%)
2	13	10	0	5	11 (85%)	1 (11%)	0	5 (50%; 100%)
3	13	9	0	3	11 (85%)	2 (22%)	0	3 (33%; 100%)
4	12	7	0	2	10 (83%)	1 (14%)	0	2 (29%; 100%)
5	11	7	0	2	8 (72%)	2 (28%)	0	2 (29%; 100%)
6	11	7	0	2	10 (91%)	1 (14%)	0	2 (29%; 100%)
7	11	11	0	6	9 (81%)	1 (9%)	0	6 (55%; 100%)
8	10	9	0	4	9 (90%)	2 (22%)	0	4 (44%; 100%)
9	10	10	0	3	8 (80%)	2 (20%)	0	3 (30%; 100%)
10	14	8	0	5	13 (93%)	1 (13%)	0	5 (63%; 100%)
avg	11.7±1.3	8.4±1.6	0	3.6±1.4	10.0±1.6 (∼85%)	1.4±0.5 (∼12%)	0	3.6±1.4(∼42%;∼10[illegible]

Table 8. Number of segments and domains generated by original and minimum networks for the 10 SF simulations. Per simulation, the number of segments and domains produced by the original network, the core network minus the segmentation gene and its incoming and outgoing regulatory links, and the minimum segment and the minimum domain networks are given. For the minimum segment network we also indicate the percentage of segments and domains it produces relative to the original network. For the minimum domain network we indicate the percentage of segments and domains it produces relative to the original network (first number) and relative to the core minus segmentation gene network (second number).

run	segm		dom		sum		overlap	
	nrg	nrc	nrg	nrc	nrg	nrc	nrg	nrc
1	7	10	13	15	17	23	3 (17.6%)	2 (8.7%)
2	11	27	9	15	16	39	4 (25.0%)	3 (7.7%)
3	7	17	9	11	12	24	4 (33.3%)	4 (16.7%)
4	12	26	11	21	15	37	8 (53.3%)	10 (27.0%)
5	11	29	6	8	14	33	3 (21.4%)	4 (12.1%)
6	11	29	6	8	15	36	2 (13.3%)	1 (2.8%)
7	8	11	17	33	15	36	7 (46.7%)	8 (22.2%)
8	10	19	12	21	18	35	4 (22.2%)	5 (14.3%)
9	10	18	9	10	14	23	5 (35.7%)	5 (21.7%)
10	9	17	13	21	17	33	5 (29.4%)	5 (15.2%)
avg	9.6±1.8	18.9±5.5	10.4±3.7	16.4±7.6	15.6±2.4	30.2±5.3	4.4±2.1 (∼28%)	5.1±3.7 (∼16%)

Table 9. Size and overlap of minimum networks for the 10 SF simulations. Per simulation, the number of genes and connections in the minimum segment and domain networks, sum of unique genes and connections in the two minimum networks together, and overlap of genes and connections between the two minimum networks are given.

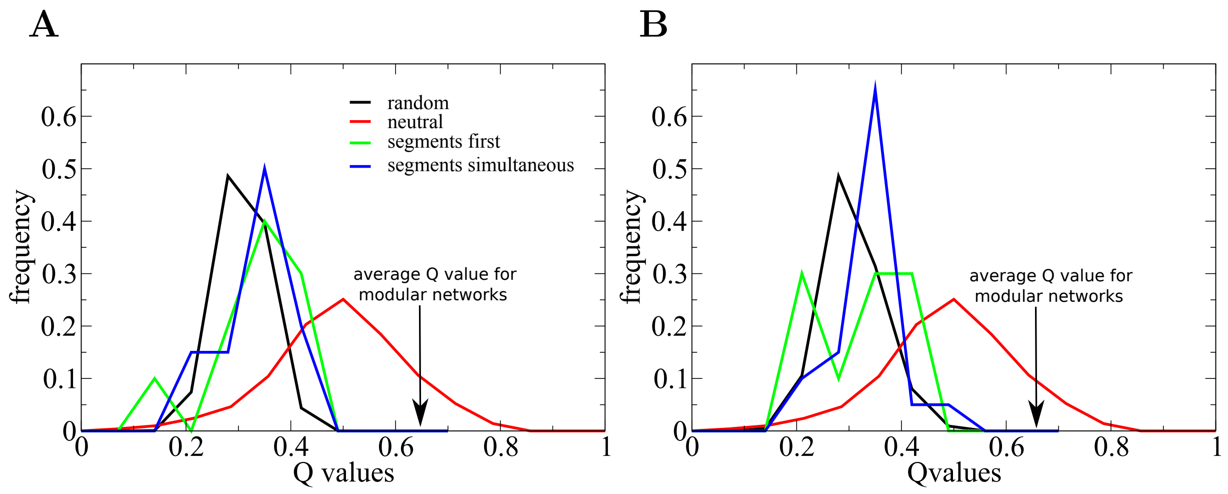

Figure 7. Q value frequency distribution for random networks, neutrally evolved networks, evolved segment first type networks, and evolved segments simultaneous type networks. Q values shown are those obtained by the walktrap method, for the leading eigenvector method similar values and distributions were obtained. For the SF and SS networks, Q values obtained for the original (**A**) and core (**B**) networks are shown. Results are also shown in Figure 3 of the main article.

value of around 0.29, neutrally evolved networks have a Q value of around 0.45, and modular networks have an average Q value of around 0.65. Thus, interestingly, neutrally evolved networks have a higher modularity score than randomly designed networks. This shows that the evolutionary dynamics (i.e. the applied mutational operators, their relative frequencies, the number of gene types that can be present) on its own, without any selection for network function and architecture increases the modularity score. Note that this is similar to previous findings in which it was shown that the high frequency of feed forward loops in biological networks can arise from neutral evolutionary dynamics and do not require any adaptive explanation [22]. It implies that modularity scores of around 0.5, which are high relative to the scores we find for random networks, do not necessarily imply a selected for, functionally relevant architectural modularity, but may be simply the result of the fact that the network is shaped through evolution.

Architectural modularity of Original and Core Networks

We see that both for segments simultaneous and for segments first type networks, and both for the original and core networks Q values lie in between those found for random and those found for neutrally evolved networks. The fact that original and core networks give similar results demonstrate that the low modularity scores are not simply the result of non-functional or redundant network components obscuring modularity.

If the type of modularity measured by these optimal Q value methods would be important to network performance, we would expect that selection would cause modularity scores of normally evolved networks to be higher than those of neutrally evolved networks. This is clearly not the case here. This suggests that no selection for this type of network modularity has occurred during evolution.

To further validate this we traced the change of Q values over evolutionary time for the two example simulations shown in Figure 3 of the main article, which each represent one of the highest Q-value scoring simulation of their type. We compared evolutionary Q value dynamics to the evolutionary changes in segment and domain numbers. Figures 8A and 8B show the evolutionary dynamics for the segments simultaneous simulation of Figure 3A of the article. We see that if we compare changes in segment and

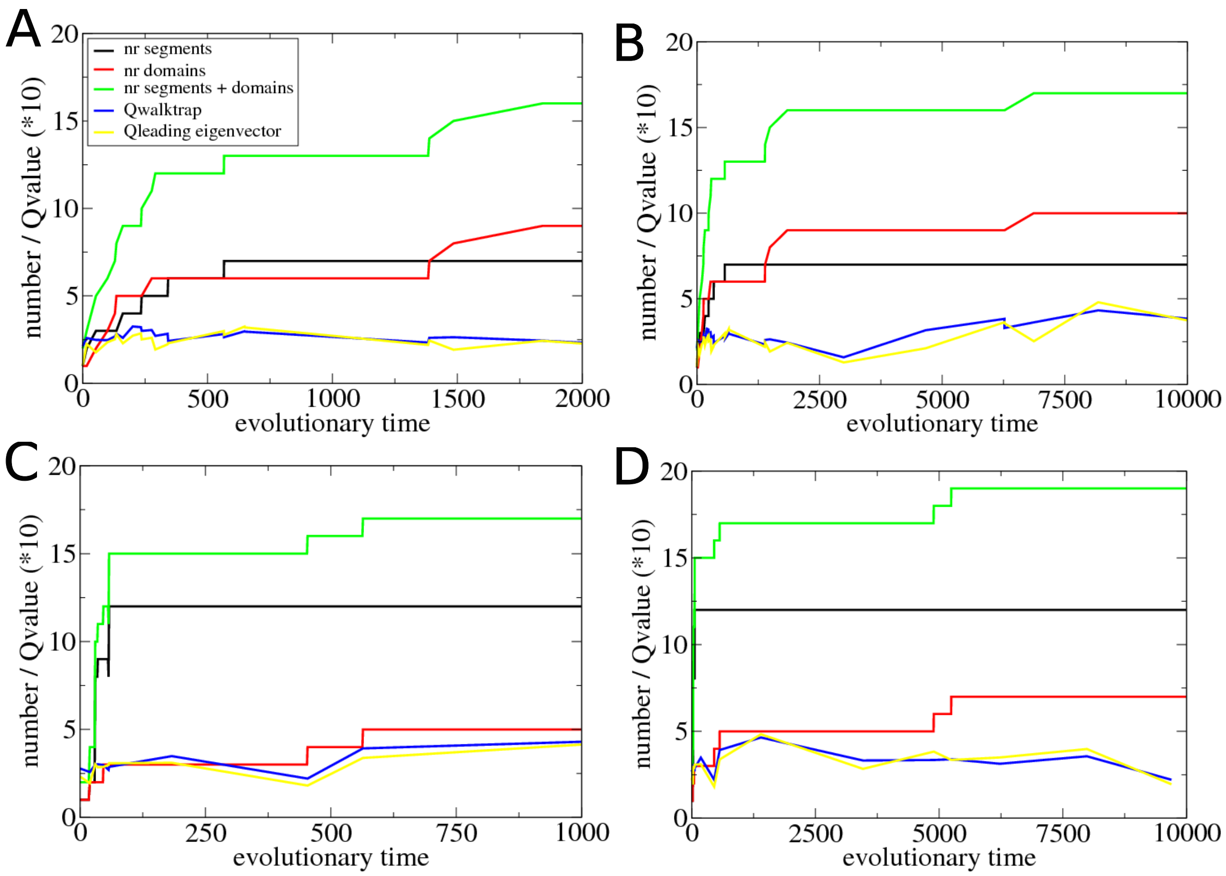

Figure 8. Change of number of segments, domains, their sum and Q-values over evolutionary time. Values are determined along the line of ancestry. Q-values are determined for the original networks, similar results were obtained for the core network. **A**, and **B** are for the segments simultaneous type simulation shown in Figure 3A of the article. **C** and **D** are for the segments first type simulation shown in Figure 3B of the article. **A** and **C** show the dynamics during the fast initial phase of evolution, **B** and **D** show dynamics over the whole period of evolution.

domain numbers, which lead to changes in fitness, with changes in Q values, no clear correlation between fitness increases and Q value increases is present. Furthermore, we also see no consistent increase of Q-values over evolutionary time. Figures 8C and 8D show similar dynamics for the segments first simulation of Figure 3B of the article. Again, we observe neither a clear correlation between fitness increases and Q-value increases nor a consistent increase in Q-value over evolutionary time. This further confirms that no selection for architectural modularity took place during evolution.

Architectural modularity under increased TFBS deletion

Table 10 shows results for default parameter settings, twofold and fivefold increased TFBS deletion rates. Compared are the success rate of simulations, the percentage of simulations applying the SF evolutionary strategy, the connectivity of original and core networks and their Q values.

setting	success rate	SF	conn orig	conn core	Qwt-orig	Qel-orig	Qwt-core	Qel-core
default	60%	33%	86.5	69.7	0.30	0.28	0.29	0.30
times 2	50%	67%	66.2	58.8	0.34	0.34	0.32	0.33
times 5	36%	0%	42.1	40.6	0.33	0.34	0.33	0.35

Table 10. Comparison of results for different TFBS deletion rates. Compared are the percentage of simulations in which successful evolution of at least 7 segments and at least 7 domains occurred, the percentage of successful simulations in which the segments first strategy evolved, the number of regulatory connections present in the original and core networks and the Q values of the original and core networks obtained with both the walktrap (Qwt) and leading eigenvector (Qel) methods.

As expected, increasing TFBS deletion rates significantly decreased the number of regulatory connections. In addition, we see that increased TFBS deletion rates significantly decrease the success rate of simulations. Interestingly, we see that for twice as large TFBS deletion rates the frequency of the functionally modular, SF strategies increases, whereas for five times as large TFBS deletion rates this frequency drops to zero. This indicates that substantially increasing TFBS deletion rates decreases rather than increases functional modularity. Finally, we see that for a twofold increase of TFBS deletion rates Q values increase from ~ 0.3 to ~ 0.33, whereas for a fivefold increase of deletion rates Q values increase only slightly more to ~ 0.34. Thus, rather than increasing TFBS deletion rates leading to modular Q values, Q values seem to saturate and remain lower than those obtained for neutrally evolved networks. Increasing TFBS deletion rates thus do not increase architectural network modularity.

As a next step, rather than starting evolution from scratch, we started simulations with the core network shown in Figure 6B of the article, and then applied the 5 times higher TFBS deletion rate. This core network is already clearly functionally modular, but does not seem architecturally modular. The idea is that by increasing TFBS deletion rate, the network connectivity will be reduced and that this might cause it to also become architecturally modular.

In Table 11 the results of these 10 simulations are shown. Perhaps surprisingly, both gene and connection numbers increase over evolution. These increases are simply the result of the used mutation rates, which cause genomes to stabilize at a certain number of genes and connections that is higher than those present in the core network we started with. However, if we compare these evolved networks with the original network from which the core was derived, or if we compare the cores of these evolved networks with the core we started with, we see that, as expected, the number of connections is significantly lower. Furthermore we see that despite this lower connectivity, no increase in architectural modularity has occurred.

run	# genes orig	# conn orig	# genes core	# conn core	Qle core	Qwt core
1	18	35	17	32	0.37	0.37
2	22	29	15	26	0.33	0.38
3	27	70	16	32	0.38	0.36
4	25	52	17	32	0.34	0.39
5	26	42	18	32	0.43	0.38
6	28	54	15	30	0.29	0.26
7	28	45	16	30	0.39	0.34
8	28	46	16	31	0.31	0.25
9	26	66	18	32	0.42	0.42
10	19	33	17	31	0.42	0.37
avg	24.7 ± 3.7	47.2 ± 13.6	16.5 ± 1.1	30.8 ± 1.9	0.37 ± 0.05	0.35 ± 0.06
original	23	57	18	36	0.40	0.37

Table 11. Results for 10 simulations in which we started with the core network shown in Figure 4 of the article. Shown are the number of genes and connections in the final evolved networks, and the minimum core networks that can be derived from these networks. Also shown are the modularity scores obtained by the two modularity algorithms for the core networks.

Functioning of alternative modularity minimum networks

As part of our alternative modularity measurement we determine minimum segment and domain networks. The minimum networks found for the SF network (shown in Figure 6B of the main article) appear to perform reasonably well at independently generating either segments or domains. Indeed, their joined dynamics and output seem to largely reproduce those of the original full network. To establish in how far this is really the case we joined the minimum segment and minimum domain networks into a single network and compared its dynamics and final developmental pattern to that of the core network (note that the output of the core network is identical to that of the original network, while the core network is simpler to analyze as the original network.

In Figure 9, left we show both the core network and the summed minimum segment and domain network of the SF network (Figure 6B of the article). We see that relative to the core network, the summed minimum segment and domain network has 1 gene and 13 connections less. In Figure 10, right we compare the final gene expression produced by the two networks. We see that the core network produces a total of 12 regularly sized segments, and a total of 4 continuous staggered expression domains using identity genes 8, 9, and 11, similar to what we saw before in Figure 4 of the article. Furthermore, we see that identity genes 8, 9 and 11, segmentation gene 5 and the identity genes 13 and 15 that covary with gene 5 have a range of different expression levels. In contrast to this, we see that the summed minimum segment and domain network produces a total of 11 segments, with the first one being considerably larger than the rest. This is similar to what we observed for the minimum segment network in isolation and thus not unexpected. Furthermore, we see that although identity genes 8, 9 and 11 are still used to generated continuous staggered expression domains, only 3 rather than 4 different staggered domains are produced. This is surprising as the minimum domain network in isolation did produce 4 different domains. Finally, we see that now all genes are expressed at the same level.

These results show that the regulatory links and gene present extra in the core but not in the minimum segment plus minimum domain network are important for segmentation and domain formation to function in combination and in a precise manner, rather than being completely redundant. Indeed, the minimum segment and domain networks are defined as the minimum set of genes and links necessary to reproduce either segments or domains to a large extent, whereas the core network is defined as the minimum set of genes and links necessary to fully reproduce the original body plan pattern with both segments and

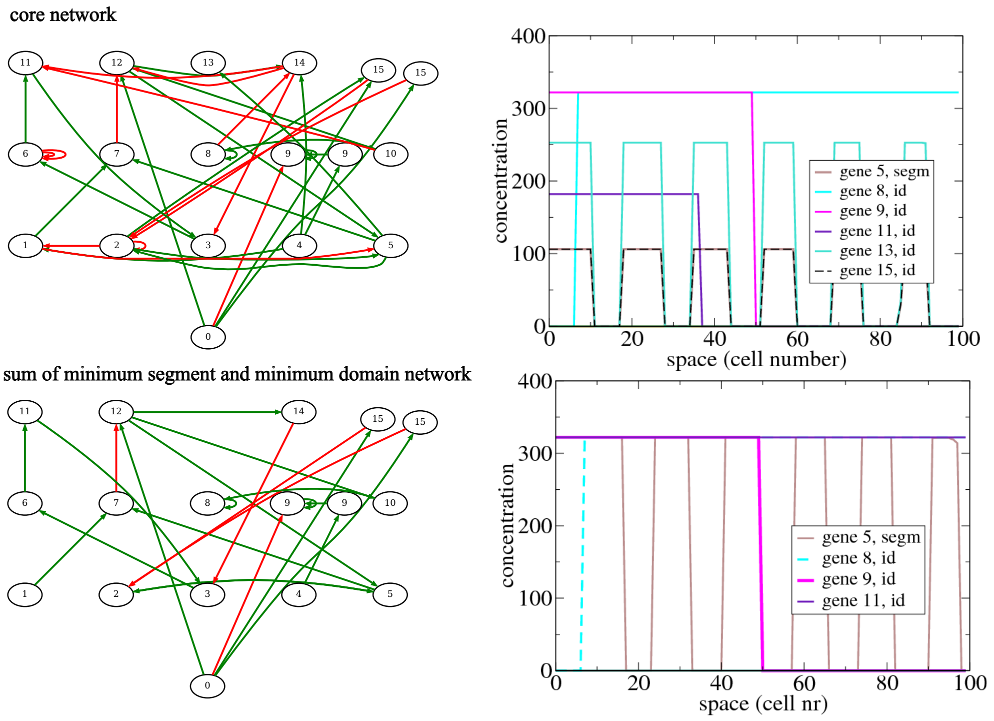

Figure 9. Top Network architecture and final gene expression pattern of the core network shown in Figure 4 of the article. **Bottom** Network architecture and final gene expression pattern of the summed minimum segment and domain networks shown in Figure 4 of the article.

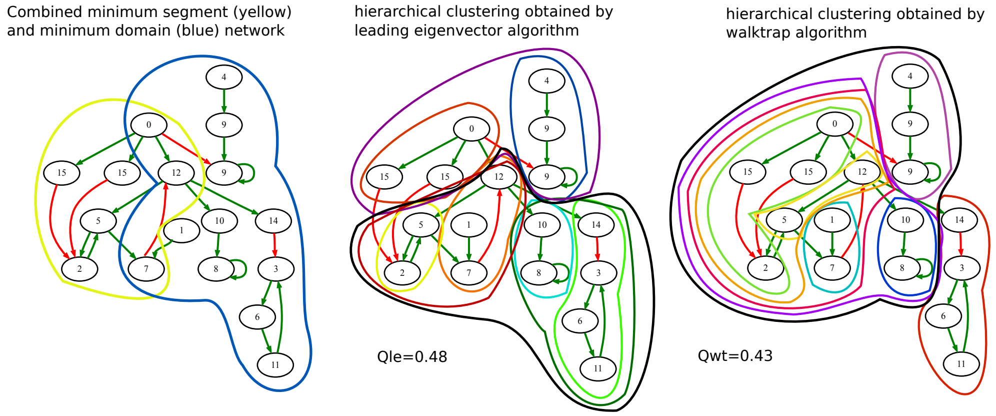

Figure 10. A Summed minimum segment and domain network, with indicated in yellow and blue the subset of genes belonging to the minimum segment and minimum domain networks respectively. **B** Again the summed minimum segment and domain network, now with colors indicated the hierarchical clusters found by the leading eigenvector method. **C** Again the summed minimum segment and domain network, now with colors indicated the hierarchical clusters found by the walktrap method.

domains.

Architectural modularity using Prior Knowledge

In Figure 10 we show the sum of the minimum segment and minimum domain networks in a different representation. On the left we indicate the genes and connections part of either the minimum segment or minimum domain network with a yellow, respectively blue enclosing line. Genes and connections lying within both the yellow and blue line are the network parts shared between the two functional minimum network modules. In the middle we use differently colored enclosing lines to indicate the hierarchical clusters obtained by the leading eigenvector algorithm. At the lowest hierarchical level, we see that 6 clusters ranging from 2 to 4 genes are formed. At the highest hierarchical level, two major clusters are formed, one containing 6 and the other containing 11 genes. We see that these architectural clusters can not be easily mapped to the functional clusters shown in the left figure.

In the right figure we show the hierarchical clusters obtained by the walktrap architectural modularity algorithm. Again we see that at the lowest level 6 clusters ranging from 2 to 4 genes are formed. However, only 3 of these 6 clusters match those found by the leading eigenvector method. At the highest level, again two major clusters are formed, one containing 4 and the other 13 genes. Note that these clusters are completely different from the ones retrieved by the leading eigenvector algorithm and again have no clear relationship with the functional modules indicated in the right figure. Finally, the modularity scores of $Q_{le} = 0.48$ and $Q_{wt} = 0.43$ are lower than those found for neutrally evolved networks.

Thus, even when using our prior knowledge that networks should generate segments and domains, and thus restricting our modularity analysis to the summed minimum segment and domain network, the architectural modularity scores fail to retrieve the modularity we clearly observed using our alternative method. We strongly suspect that this is due to the fact that the two functional modules share a small number of genes, and not just regulatory connections.

Timing and Differentiation Mechanism

In the segments simultaneous networks, a complex time transient of gene (in)activations is responsible for the formation of both domains and segments. In contrast, in the segments first networks, a minimum

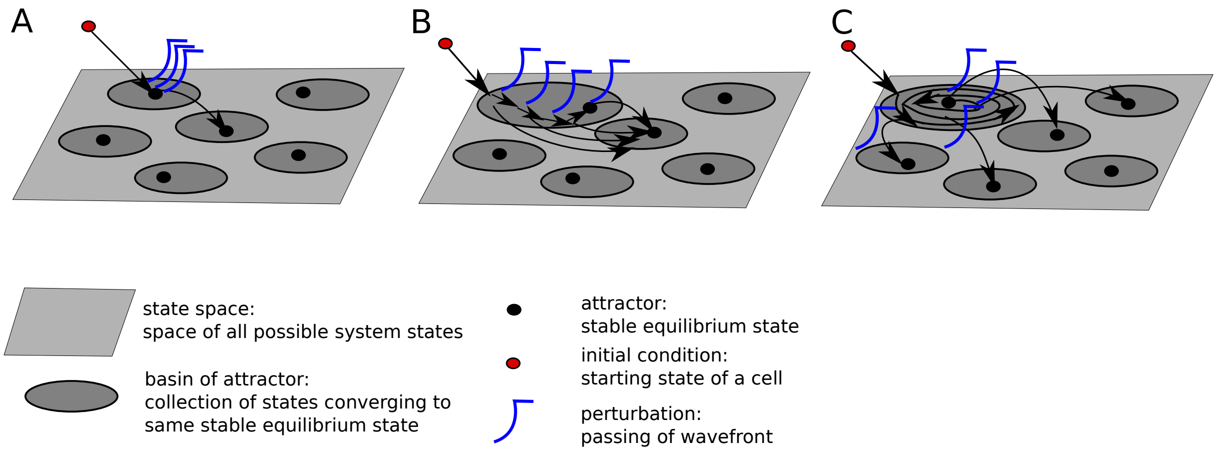

Figure 11. Transition to different attractors due to the perturbation caused by the passing of the wavefront under different types of dynamics. **A** The system converges fast from it's initial conditions to the first attractor. Independent of the time at which the morphogen wavefront passes, all cells are in this attractor, the morphogen wavefront results in the same perturbation, and the cells will switch to the same second attractor. **B** The system converges slowly from the initial condition to the first attractor. Inside the basin of attraction a slow but simple dynamics leads to convergence to the equilibrium. Dependent on the time the morphogen wavefront passes a cell, the cell will be at a different distance from this first attractor, but still upon perturbation by the passing wavefront all cells will switch to the same second attractor. **C** The system converges slowly and with a more complicated dynamics from the initial condition to the first attractor. Dependent on the time the morphogen wavefront passes a cell, the cell will be at a different distance and orientation relative to this attractor, resulting in convergence to different second attractors for different cells.

segmentation network is responsible for a clock and wavefront type segmentation process and a minimum domain network is responsible for generating the complex time transient underlying differentiation. What determines the timing of gene (in)activations that allows transformation of a temporal into a spatial gene expression pattern in these networks?

In a related study on anterior-posterior differentiation, Francois and Siggia combined a morphogen wavefront with a specially designed timer [5]. This timer gene is activated by the morphogen and has slow accumulation dynamics. As a consequence, it's local concentration depends on the time it takes for the morphogen wavefront to pass by there, thus converting the temporal wavefront into a spatial concentration gradient. In this model setup timing of gene activation thus simply depends on the timer gene and the different activation thresholds that different genes have for this timer gene.

In contrast, in our model, the timing of gene activations and inactivations are the result of distributed network properties. A gene's dynamics is dependent on the influence of genes 1-4 expressed at the start of development, the influence of the morphogen wavefront, and how overall network architecture integrates, amplifies or reduces, reroutes and delays these impacts. If, for example, gene 4 has a direct activating effect and gene 1 has a stronger but indirect inactivating effect, a gene may initially activate and later inactivate again.

The easiest way to get an understanding of this complicated network behavior is in terms of attractors, basins of attraction, time transients and perturbations. Gene regulatory networks are dynamical systems that can have one or more stable steady states or attractors. The presence of multiple attractors results from the presence of positive feedback loops in network architecture, which allow for bistability in gene expression [23–25]. For a network to produce a significant number of different domains upon passage of the

morphogen wavefront, it needs to posses two properties. First, it needs to contain sufficient independent positive feedback loops to have a considerable number of different attractors to which different cells can converge. Second, it needs to produce a long and complex enough time transient of gene activations and inactivations. To understand this latter point, first consider the opposite situation: that the network produces a short simple transient towards an attractor. The system thus converges fast and tidy to an attractor. This attractor will thus have already been reached independent of the time at which the morphogen gradient passes the cell. Once the morphogen gradient passes, (fast and tidy) convergence to a new attractor arises, which will be the same one for all cells, as they all start from the same attractor and receive the same perturbation, only on different times (Figure 12A). For different times of passing of the morphogen wavefront to produce convergence to a range of different attractors it thus is necessary that an attractor has not yet been reached before the wavefront's passage, which requires a lengthy time transient (Figures 12B and 12C), and it is necessary that the time transient towards the attractor is somewhat complicated, in the sense that perturbations at different time points in this time transient will cause the system to switch to the basin of attraction of different attractors (Figure 12C).

In general, due to the complicated dependence on distributed network properties, it is not possible to predict the produced time transient of gene (in)activations and which body regions will converge to which stable gene expression patterns from network architecture. However, it often is possible to determine the number and type of attractors from network architecture. As an example, we saw for the core network of the SS network (Figure 6A, top row) that there are 6 positive feedback loops in the core network: a positive loop between genes 1 and 14, and genes 3, 8, 11, 12 and 13 all form a positive autoregulatory loop. Due to some interdependencies these loops generated a total of 20 attractors, 10 of which are used to create the domains of the final developmental pattern. We also saw that the stable gene expression patterns of the attractors consisted of different combinations of the feedback loop genes 1-14, 3, 8, 11, 12 and 13 being expressed (Figure 6A, top row).

However, sometimes even a little more can be said based on network architecture. As an example, the minimum domain network of the SF type network (Figure 6B, top row) contains 3 positive feedback loops, a loop consisting of genes 3, 6 and 11, and positive autoregulation of genes 8 and 9. These loops produce a total of 7 attractors, and 4 of these are used to create the 4 continuous staggered domains we observe. These 4 expression domains correspond to the following 4 network attractors: 1) genes 3-6-11 and gene 9 expressed, 2) genes 3-6-11, 9 and 8 expressed, 3) genes 9 and 8 expressed and 4) gene 8 expressed. Network attractors thus indeed consist of different combinations of the positive feedback loop genes being expressed. We can easily understand how initial expression of gene 3 leads to expression of genes 3, 6 and 11. In addition, initial expression of gene 4 switches on the first gene 9, which switches on the second, autoactivating gene 9. Thus, in the anterior of the embryo, where gene 0 is only shortly expressed, genes 3, 6, 11 and 9 become stably expressed. More posteriorly, the longer expression of gene 0 causes expression of the autoactivating gene 8, via genes 12 and 10. In addition, gene 0 directly represses gene 9, and indirectly (via genes 12 and 14) represses gene 3, causing the expression of gene 9 and of genes 3, 6 and 11 to be absent from more posterior regions. Unfortunately, the precise temporal and hence spatial order in which gene 8 is switched on, gene 9 is switched off and genes 3-6-11 are switched off can not be deduced from network architecture only.

Oscillatory Segmentation Mechanism

In the SF network, the minimum segment part of the network generates oscillatory gene expression dynamics that upon passage of the morphogen wavefront is transformed into a spatially alternating stripe pattern. To get a better understanding of how exactly this works we further subdivided the minimum segment network of Figure 6B into two smaller subnetworks, which are shown in Figure 12. In the middle, we see a small subnetwork containing a positive feedback loop between genes 2 and 5 that allows for bistability in the expression of these genes. We also see that gene 0, the maternal morphogen gene, represses expression of gene 2 via gene 15, allowing it to influence which equilibrium will be approached.

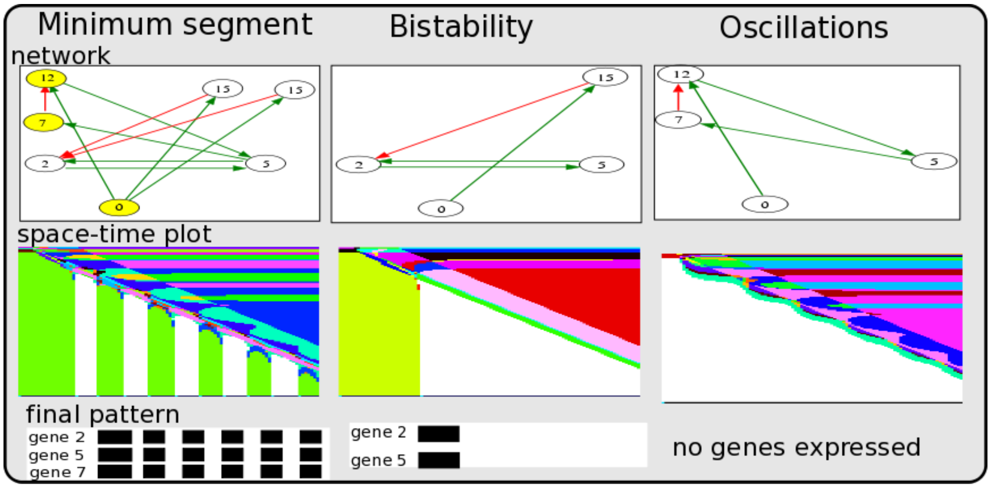

Figure 12. Network and corresponding developmental space-time plots for the minimum segment network of Figure 6B of the article (left), the subnetwork responsible for generating bistability (middle) and the subnetwork responsible for generating oscillations (right)

At the anterior end of the embryo, where gene 0 is only shortly expressed, genes 2 and 5 become stably expressed. In contrast, in the rest of the embryo, where gene 0 is expressed longer, neither gene 2 nor gene 5 becomes expressed.

On the right we see another small subnetwork containing the combination of a negative feedback loop between genes 5, 7 and 12 and a positive link coming from the maternal morphogen gene 0. Note that because gene 0 is persistently expressed until the wavefront has passed by this is equivalent to a positive feedback loop. We see in the developmental space-time plot that this combination produces oscillations. When the wavefront has passed by and the positive feedback of gene 0 disappears the oscillations stop and gene expression ceases.

Together, the bistability and oscillator network motifs enable the minimum segment networks to transform a temporally oscillating into a spatially alternating expression pattern: depending on the phase of the oscillation when the wavefront passes by expression of gene 5 is either high or low and cells will either stably express or not express genes 2 and 5.

Movies

Supplementary movies can be found at:
http://www-binf.bio.uu.nl/khwjtuss/1DAnimalMovies

The movie **S1.mpg** shows the spatiotemporal dynamics of all 16 gene types during development of the same fit individual described in Figure 3A in the main manuscript. Gene expression levels (protein concentrations) are encoded in gray scales, white meaning high, Gray intermediate and black zero gene expression. The 16 gene types are ordered in 4 rows of 4 genes, running from left top to right bottom. Per gene, the anterior of the embryo is to the left and the posterior to the right.

In the movie one can clearly see the expression of the maternal morphogen wavefront gene (row 1, column 1) going from high to zero levels in an anterior to posterior manner. Furthermore we see that genes 1 and 14 (row 1, column 1 and row 4, column 3) become expressed in a small anterior region and also in the posterior one third of the embryo. Next, we see that genes 2 and and 10 (row 3, column 4 and

row 3, column 3) become expressed in an anterior region covering approximately one third of the embryo. The segmentation gene (gene 5, row 2, column 2) is initially nowhere expressed, but early in development it reaches a global high expression level. After this, the expression of gene 5 becomes restricted to those regions where gene 14 or gene 15 are expressed and gene 8 is not expressed.

We also see that genes 6 and 15 (row 2, column 3 and row 4, column 4) become expressed in a similar sized domain as genes 2 and 10 but located more posteriorly. Expression of gene 7 (row 2, column 4) is established everywhere in the embryo except for a small region close to the middle. During development gene 8 (row 3, column 1) becomes expressed in two small anterior regions and a larger posterior region. We see that gene 9 (row 3, column 2) becomes expressed in the posterior one third of the embryo, whereas expression of gene 11 (row 3, column 4) arises in the posterior region of the embryo except for the most posterior part. Gene 12 (row 4, column 1) becomes expressed in all but the most posterior part of the embryo. Finally, gene 13 (row 4, column 2) becomes expressed in a large anterior region.

Movie **S2.mpg** shows the same spatiotemporal gene expression dynamics as the previous movie, but in a different way. The expression levels of all 16 genes as a function of their position along the anterior posterior axis of the embryo are drawn in a single plot which changes over time. We can see the same spatiotemporal gene expression dynamics as described above. In addition, we clearly see how the passing of the wavefront freezes the local complex time transient into a stable expression pattern.

The movie **S3.mpg** shows the spatiotemporal dynamics of all 16 gene types during development of the same fit individual described in Figure 3B in the main manuscript. The format is the same as in the movie **S1.mpg**.

Again, we clearly see the progression of the maternal morphogen wavefront (row 1, column 1). Furthermore, we can see how temporal oscillations in the expression level of the segmentation gene (row 2, column 2) get frozen into alternating segments of high and zero expression of that gene. We also see that genes 2 (row 1, column 3), 7 (row 2, column 4), 13 (row 4, column 2) and 15 (row 4, column 4) have a spatiotemporal expression pattern that co-varies with that of the segmentation gene. We furthermore see that during development an anterior expression domain of genes 3, 6, and 11 covering approximately 4/9-th of the embryo is established. Gene 9 (row 3, column 2) becomes expressed in a region occupying the anterior half of the embryo. Finally, gene 8 (row 3, column 2) becomes expressed in all but the most anterior part of the embryo.

Movie **S4.mpg** shows the same spatiotemporal gene expression dynamics as the previous movie, now plotting all gene expression levels as a function of their position along the anterior posterior axis of the embryo are drawn in a single plot which changes over time. In this movie we can clearly see how the oscillations of the segmentation gene and the genes 2, 7, 13 and 15 are transformed by the passing maternal morphogen gradient into an alternating on/off segmentation pattern. In addition we can clearly see the formation of the relative to one another staggered expression domains of genes 3-6-11, gene 8 and gene 9.

References

1. Toffoli T, Margolus T (1987) Cellular Automata Machines: A New Environment for Modeling. MIT Press, Cambridge, Mass.

2. Francois P, Hakim V, Siggia ED (2007) Deriving structure from evolution: metazoan segmentation. Mol Syst Biol 3: 154.

3. Fujimoto K, Ishihara S, Kaneko K (2008) Network evolution of body plans. PLoS One 3: e2772.

4. Walczak AM, Tkacik G, Bialek W (2010) Optimizing information flow in small genetic networks. ii: Feed-forward interactions. Phys Rev E 81: 041905.

5. Francois P, Siggia ED (2007) Predicting embryonic patterning using mutual entropy fitness and in silico evolution. Development 137: 2385-2395.

6. Davis GK, Patel NH (1999) The origin and evolution of segmentation. Trends Cell Biol 9: M68–M72.

7. Peel A, Akam M (2003) Evolution of segmentation: rolling back the clock. Curr Biol 13: R708–R710.

8. Peel A (2004) The evolution of arthropod segmentation mechanisms. Bioessays 26: 1108–1116.

9. Tautz D (2004) Segmentation. Dev Cell 7: 301–312.

10. Liu PZ, Kaufman TC (2005) Short and long germ segmentation: unanswered questions in the evolution of a developmental mode. Evol Dev 7: 629–646.

11. Rivera AS, Weisblat DA (2009) And Lophotrochozoa makes three: Notch/Hes signaling in annelid segmentation. Dev Genes Evol 219: 37–43.

12. Kashtan N, Alon U (2005) Spontaneous evolution of modularity and network motifs. Proc Natl Acad Sci U S A 102: 13773-13778.

13. Crombach A, Hogeweg P (2007) Chromosome rearrangements and the evolution of genome structuring and adaptability. Mol Biol Evol 24: 1130–1139.

14. Crombach A, Hogeweg P (2008) Evolution of evolvability in gene regulatory networks. PLoS Computational Biology 11: 21000112.

15. Draghi J, Wagner G (2008) Evolution of evolvability in a developmental model. Evolution 62: 301-315.

16. Draghi J, Wagner G (2009) The evolutionary dynamics of evolvability in a gene network model. J Evol Biol 22: 599-611.

17. Wagner A (2005) Robustness, evolvability, and neutrality. FEBS Lett 579: 1772–1778.

18. Wagner A (2008) Robustness and evolvability: a paradox resolved. Proc Biol Sci 275: 91–100.

19. Newman M (2006) Modularity and community structure in networks. Phys Rev E 74: 036104.

20. Newman M (2006) Finding community structure in networks using the eigenvectors of matrices. Proc Natl Acad Sci USA 103: 8577-8582.

21. Pons P, Latapy M (2006) Computing communities in large networks using random walks. J Graph Algorithms Appl 10: 191-218.

22. Cordero O, Hogeweg P (2006) Feed-forward loop circuits as a side effect of genome evolution. Mol Biol Evol 23: 1931-1936.

23. Thomas R (1981) On the relation between the logical structure of systems and their ability to generate multiple steady states or sustained oscillations. Springer series in Synergetics 9: 180-193.

24. Thomas R, Kaufman M (2001) Multistationarity, the basis of cell differentiation and memory. i. structural conditions of multistationarity and other nontrivial behavior. Chaos 11: 165-179.

25. Thomas R, Kaufman M (2001) Multistationarity, the basis of cell differentiation and memory. ii. logical analysis of regulatory networks in terms of feedback circuits. Chaos 11: 165-179.
